# Potential Impact of Microbial Variations After Peri-Implantitis Treatment on Peri-Implant Clinical, Radiographic, and Crevicular Parameters: A Systematic Review

**DOI:** 10.3390/dj12120414

**Published:** 2024-12-17

**Authors:** Federica Di Spirito, Massimo Pisano, Maria Pia Di Palo, Flora Salzano, Antonio Rupe, Antonino Fiorino, Carlo Rengo

**Affiliations:** 1 Department of Medicine, Surgery and Dentistry, University of Salerno, Via S. Allende, 84081 Salerno, Italy; pisano.studio@virgilio.it (M.P.); mariapia140497@gmail.com (M.P.D.P.); flo.salzano@gmail.com (F.S.); antoniorupe@virgilio.it (A.R.); 2 Department of Neuroscience, Reproductive Science and Dentistry, University of Naples Federico II, 80131 Naples, Italy; fiorinodr.antonino@gmail.com

**Keywords:** peri-implant disease, peri-implantitis, peri-implant mucositis, peri-implantitis treatment, microbiome, microorganisms

## Abstract

**Objectives:** This systematic review evaluated concomitant trends in microbial (total biofilm load and pre-dominant pathogens’ counts) and clinical, radiographic, and crevicular variations following (any) peri-implantitis treatment in partially vs. totally edentulous, systemically healthy, non-smoking adults and compared them to peri-implant mucositis treated sites. **Methods:** The study protocol, compliant with the PRISMA statement, was registered on PROSPERO (CRD42024514521). Findings from six randomized controlled trials (RCTs), evaluated through the ROBINS-2 tool, were qualitatively synthesized. **Results:** No data concerning total edentulism and treated peri-implant mucositis sites were retrieved from the included RCTs. Instead, as expected, in the partially edentulous subjects, peri-implantitis treatments effectively provided biofilm control, although Plaque Index (PI) tended to increase again over time. Notably, Bleeding on Probing (BoP) rose slightly after treatment but decreased markedly by three months, indicating, at least, a partial resolution of the infective-inflammatory process. Probing Depth (PD) showed a slower but consistent improvement throughout. Despite a return of PI levels by twelve months, BoP and PD continued to improve, underscoring the successful long-term outcomes of peri-implantitis treatment. Over time, variations in PI did not consistently reflect changes in predominant pathogenic species, especially at the 1-month follow-up; BoP aligned with predominant pathogens rather than total microbial biofilm load at the 1- and 3-month follow-ups, and PD did the same at the 3- and 6-month follow-ups, likely affecting peri-implantitis-associated microbiota. No data concerning crevicular parameters were retrieved in the included RCTs, and the extracted radiographic outcomes were not comparable. **Conclusions:** The impact of the microbial variations after peri-implantitis treatment on peri-implant clinical parameters highlight the critical role of dysbiosis, rather than total microbial load, in influencing inflammation and tissue destruction, emphasizing the need for targeted approaches to manage persistent pathogens and improve treatment efficacy.

## 1. Introduction

Dental implant-supported restorations are currently a common treatment for the rehabilitation of partially or totally edentulous subjects [1]. The increasing number of dental implant-supported restorations placed annually has led to an escalation in diagnosing peri-implant diseases (peri-implant mucositis and peri-implantitis) [1]. Peri-implantitis represents the most frequently encountered complication in the later stages of dental implant therapy, impacting an estimated 15–57% of individuals and 8–28% of dental implants [1]. If left unmanaged, this condition may progress to implant failure, reported to affect approximately 8% of patients and 4% of implants [2].

The 2017 World Workshop defined peri-implantitis as a plaque-associated pathological condition of the tissues around implants, characterized by peri-implant mucosa inflammation and progressive bone loss, and simplified the radiographic bone level assessment than the previous diagnostic criteria, requiring a bone loss of >2 mm in the first year and/or >0.2 mm afterward [3]. The current diagnosis of peri-implantitis requires bleeding and/or suppuration on gentle probing, probing at depths of ≥6 mm, and a bone level of ≥3 mm apical to the most coronal plane of the intrabony portion of the implant at any time [3].

Instead, as stated in the 2017 Workshop from an etiological perspective, peri-implantitis, similar to periodontitis, is an infectious and inflammatory condition driven by the host’s immune response to oral dysbiosis [4]. This imbalance in the oral microbiome, influenced by genetic and environmental factors such as diet, oral hygiene, stress, alcohol and smoking habits, medications (e.g., antibiotics, corticosteroids), and various systemic or oral diseases [5] beyond dentition, can contribute to the development of microbiota-associated oral conditions, including peri-implantitis [5,6].

Socransky et al. [7] classified the predominant subgingival biofilm pathogens into five major complexes in 1998: red, orange, green, yellow, and purple. Additionally, certain microorganisms were categorized as “outliers” due to their limited interactions with the bacteria within these complexes. Compared to healthy periodontal sites, periodontitis-associated microbiota demonstrates an increased overall microbial load and a higher prevalence of key periodontal pathogens, especially those from the red complex (*Porphyromonas gingivalis*, *Tannerella forsythia*, and *Treponema denticola)*, along with *Fusobacterium nucleatum*, *Prevotella intermedia*, and *Aggregatibacter actinomycetemcomitans*. A general shift from aerobic Gram-negative bacteria to strictly anaerobic Gram-negative bacteria is also observed in periodontitis [8,9].

Anaerobic bacteria are similarly dominant in peri-implant sites, particularly when Probing Depth (PD) exceeds 5–6 mm, signaling the need for clinical intervention [10]. The microbial profile in peri-implantitis includes a broader diversity [11,12,13], with a slightly increased abundance of bacteria from the red and orange complexes (e.g., *Prevotella intermedia* and *Prevotella nigrescens*) compared to periodontitis [6,11,14]. Indeed, higher levels of *Prevotella intermedia*, *Prevotella nigrescens*, *Porphyromonas gingivalis*, *Treponema denticola*, *Tannerella forsythia*, and *Aggregatibacter actinomycetemcomitans* have been detected in peri-implantitis sites relative to those affected by periodontitis [6,11,14].

Hence, microbial load reduction and dysbiosis reversal inducing favorable changes in the peri-implantitis-associated microbiota are the main goals of peri-implantitis treatment, beyond host immune–inflammatory modulation to achieve inflamed soft tissue healing, PD (≤5 mm) reduction, and bone loss halt [4,15].

Accordingly, several peri-implantitis treatments have been proposed to achieve microbial load reduction and dysbiosis reversal [16,17,18], including mechanical [19], chemical [16,17,18], and physical [4,20,21] decontamination methods, through surgical, non-surgical, or combined approaches [4,16,17,18,19,20,21,22,23,24].

Although several studies investigated the clinical, biochemical, and radiographic outcomes of different peri-implantitis treatments [12,13,16,17,19,20,22], and peri-implantitis management is overall based on biofilm control [17], later secured by supportive treatment [22], the associated microbiological downsides and related clinical implications remain unclear.

The hypothesis is that the microbiota variations (both in total biofilm microbial load and predominant pathogens’ count) after treatment will be associated with improvements in clinical, radiographic, and crevicular peri-implant parameters. If this hypothesis is confirmed it should provide insights into the relationship between microbiota variations and tailored, targeted, and timed peri-implantitis treatment, offering potential parameters for evaluating the effectiveness of peri-implantitis therapies.

Given the dysbiosis role in peri-implantitis progression and the several peri-implantitis treatments available, understanding the potential impact of microbial variations over time after peri-implantitis treatment on peri-implant clinical, radiographic, and crevicular parameters is essential for evaluating tailored, targeted, and timed peri-implantitis site therapeutic strategies.

Therefore, the present systematic review investigated the potential impact of peri-implantitis-associated microbiota variations (total biofilm microbial load and predominant pathogens’ counts) on clinical, radiographic, and crevicular peri-implant parameters recorded after (any) peri-implantitis treatment in partially and totally edentulous, systemically healthy, non-smoking adults. The secondary aims were to compare findings at treated peri-implantitis sites in subjects with partial vs. total edentulism and treated peri-implant mucositis sites.

## 2. Materials and Methods

### 2.1. Study Protocol

The study protocol was drafted under the PRISMA (Preferred Reporting Items for Systematic Reviews and Meta-Analyses) statement [25] and registered (CRD42024514521) in the PROSPERO register for systematic reviews before the electronic and manual literature search began.

The research question was as follows: “What is the potential impact of microbial variations in total biofilm load and predominant pathogens’ counts on clinical, radiographic, and crevicular parameters after (any) peri-implantitis treatment in partially and totally edentulous, systemically healthy, non-smoking adults?”.

The PICO framework [26] was employed for structuring clinical questions, as follows:

- Population (P): partially and totally edentulous, systemically healthy, non-smokers, ≥ 18 years, diagnosed with (at least one) treated site diagnosed with peri-implantitis according to previous and current criteria [3,27];
- Intervention (I): all surgical and non-surgical approaches for the treatment of peri-implantitis in combination with sampling and analysis of supra-/sub-mucosal peri-implantitis-associated microbiota before (baseline) and after treatment;
- Comparison (C): data from partially vs. totally edentulous subjects and from treated sites with peri-implant mucositis vs. peri-implantitis;
- Outcomes (O): variations in microbial total biofilm load and predominant pathogen counts and in clinical, radiographic, and crevicular peri-implant parameters after (any) peri-implantitis treatment.

### 2.2. Search Strategy

Two reviewers (C.R., A.F.) independently searched three electronic databases, namely Web of Science, MEDLINE/PubMed, and Scopus, to find studies published in English from 20 years ago to 5 July 2024. The 20-year timeframe was defined based on the rapid evolution of dental implants [1], the novel treatments available for related diseases (e.g., regenerative or antimicrobial therapies) [16,17,18], and the advancements in the knowledge of peri-implantitis microbial cluster and analysis techniques [14].

The keywords were (“peri-implant disease” OR “peri-implantitis” OR “implant failure” OR “peri-implant failure”) AND (microorganisms OR “oral dysbiosis” OR bacteria OR microbiota OR microbiome OR viruses OR virus OR fungi OR fungus) AND (therapy OR treatment OR “surgical treatment” OR “non-surgical treatment” OR approach OR approaches OR “debridement”) and were combined using Boolean operators.

The following filters were applied according to their availability for each database to refine the electronic search to the original article in the English language:

- Web of Science and Scopus: “languages”, English; “document type”, article.
- MEDLINE/PubMed: “article language”, English; “article type”, not review and not systematic review.

The same authors (C.R., A.F.) conducted a manual search by screening the reference lists of the studies selected by the electronic search in order to obtain additional potential records.

### 2.3. Study Selection

Two authors (F.D.S., M.P.D.P.) conducted an independent selection of studies. All titles retrieved from the electronic search in the databases and register underwent screening, duplicate entries were removed, and pertinent abstracts were assessed. In instances of discordance, a third author (A.R.) participated in the study selection process, and any uncertainties were resolved through discussion to make the final decision. Therefore, at least two authors achieved consensus on each study selection step for each record screened. Subsequently, the same two reviewers independently obtained and scrutinized the full texts of the potentially eligible titles/abstracts. In cases where the full text was unavailable, the authors of the respective studies were contacted.

This identical process of study selection was applied to records identified through a manual search in the reference lists of included studies.

### 2.4. Eligibility Criteria

Inclusion criteria: the present systematic review focused on case–control studies, cohort studies, and randomized clinical trials published in English in the last 20 years, encompassing adult individuals (≥18 years) who were systemically healthy, non-smoking, and either partially or completely edentulous. Participants were required to have at least one peri-implantitis site, with a concomitant assessment of peri-implantitis-associated microbiota and clinical, radiographic, and crevicular peri-implant parameters both before and after treatment. No limitations were imposed regarding the publication date of articles, participants’ gender, types of peri-implantitis treatment, or methods utilized for microbiological sampling and analysis. The total peri-implant microbiological load and/or the predominant pathogens’ count was required to be in numerical quantitative value independent of the total microbial load.

Exclusion criteria: animal studies, in vitro studies, reviews (any type), pre-clinical studies, case-reports, and case series studies; studies and records not written in English not; investigations involving participants of <18 years of age or smokers; oncological patients, individuals who had undergone prior radiotherapy or chemotherapy treatment, immunocompromised subjects, or patients who had received immunosuppressive therapy or antibiotics and/or corticosteroids within the preceding three months (as these medications may precipitate oral dysbiosis); pregnant individuals or those with a history of uncontrolled periodontitis; studies focusing on extraoral, zygomatic, or orthodontic implants; investigations evaluating diverse parameters such as radiographic bone level, Probing Depth (PD), Bleeding On Probing (BoP), and suppuration for peri-implantitis diagnosis [3,27]; and investigations evaluating the total peri-implant microbiological load and/or the predominant pathogens’ count as a non-quantitative numerical value or dependent on the total microbial load (e.g., predominant pathogen counts reported as a percentage of the total microbial load or where microorganisms were considered to be positive when the microbial load exceeded an established cut-off per site).

### 2.5. Data Extraction and Collection

Two authors (F.D.S., M.P.D.P.) conducted autonomous data extraction and collection utilizing a specified template in accordance with the prescribed models for intervention reviews of RCTs [25]. In instances of discordance, a third author (A.F.) participated in the data extraction and collection process, and any uncertainties were resolved through discussion to make the final decision. Therefore, at least two authors achieved consensus on each data extraction and collection step for each record included. Only data conforming to the predetermined eligibility criteria were subjected to extraction and collection. Furthermore, data pertaining to partially edentulous individuals with at least one treated peri-implantitis site were independently extracted and collected, distinct from data concerning totally edentulous individuals (restored with full-arch dental implant-supported prostheses with at least one treated peri-implantitis site), as well as treated peri-implant mucositis.

No efforts were undertaken to establish contact with the authors of the included studies for the acquisition or validation of data.

The following data were extracted and collected from each record.

- Studies: author (first), year and journal of publication, study design and quality, funding (if any);
- Population: number of participants, mean age, and gender ratio; number of treated peri-implantitis sites and of supported restoration, dental implant design, and position;
- Intervention: peri-implantitis treatment approach (surgical or non-surgical), procedure(s), number of sessions, timings, and the methods and timings of microbiological sampling and analysis;
- Outcome(s): variations in peri-implant total biofilm microbial load and predominant pathogens’ counts and of clinical, radiographic, and crevicular peri-implant parameters after (any) peri-implantitis treatment.

### 2.6. Data Analysis

Data concerning study participants, peri-implantitis treatment, and relevant outcomes were qualitatively synthesized through Microsoft Excel software 2019 (Microsoft Corporation, Redmond, WA, USA).

### 2.7. Risk Assessment

The RCTs incorporated into the present investigation underwent evaluation utilizing the Revised Cochrane Risk-of-Bias 2 tool for randomized trials (RoB 2) [28], accessed online at no cost on 24 February 2024. This assessment was conducted by three independent reviewers (M.P.D.P., A.R., C.R.).

The RoB 2 tool assessed bias arising from the randomization process, assignment concealment and intervention adherence, missing outcome data, outcome measurement, and selection of the reported results [29].

Judgment of risk was categorized as follows: “low” if all domains demonstrated low risk of bias, “unclear” if at least one domain had unclear risk but no domain exhibited high risk, and “high” if multiple domains were unclear or if at least one domain presented high risk of bias [29].

## 3. Results

### 3.1. Study Selection and Description

A total of 1570 records were identified through an electronic database search: 635 from Scopus, 579 from Web of Science, and 356 from PubMed/MEDLINE. After removing 600 duplicate entries, 970 titles and abstracts were screened. Of these, 787 records were excluded as they were not relevant to the objectives of this systematic review.

The remaining 183 full-text articles were retrieved without requiring contact with the authors. A thorough assessment of these articles was conducted to evaluate their eligibility. Ultimately, 178 studies were excluded for the following reasons: in vitro or animal-based research (*n* = 64); the absence of adult participants or peri-implantitis treatment (*n* = 33); the inability to extract microbiological data or lack of such analysis (*n* = 21); the absence of data from partially or fully edentulous adult non-smokers (*n* = 18); narrative or systematic reviews (*n* = 14); bacterial counts not reported as absolute values (*n* = 8); case reports or case series (*n* = 6); studies older than 20 years (*n* = 5); pilot or protocol studies (*n* = 2); and non-English publications (*n* = 1).

The process of study selection via databases led to the inclusion of five RCTs [30,31,32,33,34] in the present systematic review.

A similar procedure was applied during the manual search, in which the reference lists of these five studies [30,31,32,33,34] were screened.

A total of 166 references were identified through the manual search, with 15 duplicates removed. The remaining 151 titles and abstracts were screened, resulting in the exclusion of 144 records due to irrelevance to the objectives of this systematic review.

The full texts of the seven remaining articles were retrieved without requiring author contact and were thoroughly evaluated for eligibility. Six studies were excluded for the following reasons: they were not randomized controlled trials (RCTs) (*n* = 1); microbiological analysis data were either unavailable or not performed (*n* = 4); or data from non-smoking subjects could not be extracted (*n* = 1).

The manual search process ultimately identified one RCT [35] eligible for inclusion in this systematic review.

**Figure 1:**
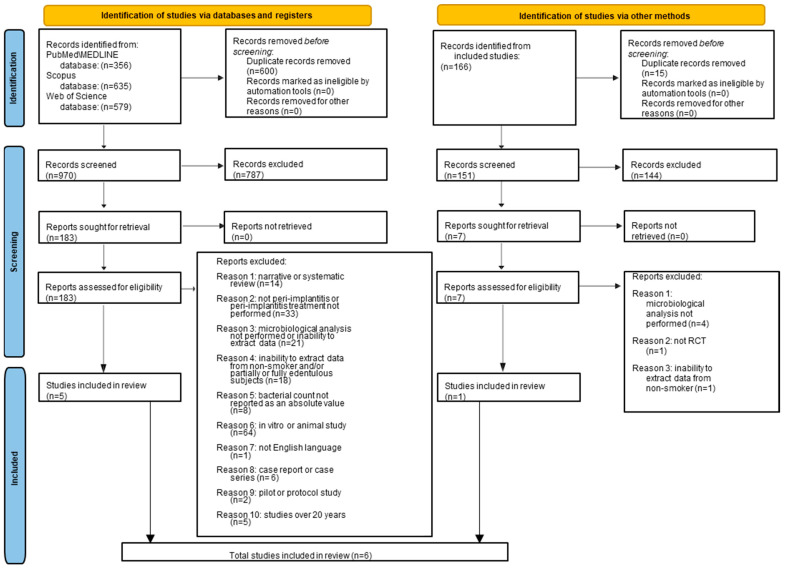
PRISMA 2020 flowchart for new systematic reviews, including the study selection process via databases, registers, and other methods.

Following both electronic and manual searches, a total of six RCTs [30,31,32,33,34,35] were included in the final analysis (Figure 1).

A total of six RCTs [30,31,32,33,34,35] that met the eligibility criteria were included and processed for data extraction, data synthesis, and quality assessment.

The study population comprised 164 partially edentulous, systematically healthy, non-smoking adult subjects with a total of 186 treated peri-implantitis sites. No data on completely edentulous adult subjects or on treated peri-implant mucositis sites were found.

The mean age and gender ratio of the population were reported in three studies [30,33,34]. The weighted mean age was 57.06 ± 23.95 years with a M:F gender ratio of 2.24:1.

The characteristics of the dental implants were reported in one study [36] (*n* = 40 Nobel Biocare^®^ dental implants with rough surfaces). The dental implant (bone/tissue level), the type of implant abutment, and the interim period after implant placement were not defined in any study.

One study [33] reported the total number of prosthesis restorations: fixed partial prosthesis, *n* = 128, and single crown, *n* = 72.

In four studies [30,32,33,34,35], non-surgical peri-implantitis treatment was performed on a total of 146 peri-implantitis sites: NSMD (*n* = 23 peri-implantitis sites treated); NSMD plus chlorhexidine plus local antibiotics plus diode laser (*n* = 20); NSMD plus chlorhexidine plus local antibiotics plus diode laser plus aPDT (*n* = 20); NSMD plus air polishing plus diode laser (*n* = 20); NSMD plus air polishing plus diode laser plus aPDT (*n* = 20); Er: YAG laser (*n* = 13); NSMD plus probiotics (*n* = 11); NSMD plus air polishing (*n* = 10); and NSMD plus air polishing plus probiotics (*n* = 9).

In one study [31], surgical peri-implantitis treatment was performed on a total of 40 peri-implantitis sites, in particular, SMD plus local CHX and CHX mouth rinses for 2 weeks (*n* = 20) and SMD plus local CHX plus diode laser plus aPDT plus CHX mouth rinses for 2 weeks (*n* = 20).

The study [31] in which the authors performed the surgical treatment of peri-implantitis was the only one that stated that the prosthetic structures were not removed in order to perform the treatment. The other studies [30,32,33,34,35] did not detail this aspect of treatment.

Peri-implantitis treatment was performed in one session in five studies [30,31,32,33,34], with a total of 133 peri-implantitis patients treated; in one study [35] in two sessions (*n* = 40); and in one study [32] in three sessions (*n* = 13) at baseline, after 2 and 4 weeks.

For each RCT [30,31,32,33,34,35], information was collected on the first author, year and journal of publication, study design, reference, quality and funding sources, study population, peri-implant site characteristics, and peri-implantitis treatment performed. The characteristics of the population included the sample size, the mean age and age range of the participants, and the gender ratio. For the peri-implant sites, the data included the total number of sites, the number of peri-implantitis sites and details of implant design and type, the level of implant placement, whether in soft tissue or bone, and the type of abutment used. Information on the supported restoration included the type and number of restorations and the mean time elapsed since implant placement. The treatment of peri-implantitis was specified, indicating the type of treatment, the removal of the prosthesis, and the number of treatment sessions performed.

These extracted and collected data are summarized in Table 1.

### 3.2. Outcome Reporting

Peri-implantitis-associated microbiota variations (total biofilm microbial load and predominant pathogens’ counts) and clinical, radiographic, and crevicular peri-implant parameters recorded at short-term (1 and 3 months) and long-term (6 and 12 months) follow-ups after (any) peri-implantitis treatment in partially and totally edentulous, systemically healthy, non-smoking adults were extracted and collected from the six RCTs [30,31,32,33,34,35] included in the present systematic review.

Table 2 summarizes the data of the peri-implant total microbial load and predominant pathogens’ counts along with peri-implant clinical parameters at treated peri-implantitis sites and full mouth clinical parameters scores, before and after treatment.

### 3.3. Peri-Implantitis-Associated Microbiota Variations over Time After Peri-Implantitis Treatment

Microbiological peri-implant data were obtained from 164 subjects involved in the RCTs included with 186 treated peri-implantitis sites. No data were retrieved on total edentulism and treated peri-implant mucositis sites.

Microbiological analyses conducted in the RCTs were PCR (unspecified target) [30], RT-PCR (unspecified target) [35], bacterial cultures [31], RT-PCR (unspecified target) [33], RT-PCR (target: DNA and 16s RNA) [34], and unspecified in one study [32].

#### 3.3.1. Total and Anaerobic Load

The total peri-implant biofilm microbial loads, expressed as total bacterial count, were reported in a study [33] at 22 peri-implant sites at baseline, one-month follow-up, and three-month follow-up and were 9.18 log CFU/mL, 9.36 log CFU/mL, and 9.14 log CFU/mL, respectively.

The peri-implant anaerobic load was recorded before treatment at 65 peri-implantitis sites [31,32] measuring 5.51 log CFU/mL. After three months, it decreased to 4.58 log CFU/mL at 65 treated peri-implantitis sites [31,32], and after six months to 4.53 log CFU/mL at 65 treated peri-implantitis sites [31,32].

#### 3.3.2. Predominant Pathogens

At the baseline (all the 186 treated peri-implantitis sites [30,31,32,33,34,35]), predominant pathogen counts varied, with *Fusobacterium nucleatum* (*F. nucleatum*) showing the highest count at 6.84 log CFU/mL, followed by *Campylobacter rectus* (*C. rectus*) (6.02 log CFU/mL), *Peptostreptococcus micros* (*P. micros*) (5.99 log CFU/mL), and *Eikenella corrodens* (*E. corrodens*) (5.04 log CFU/mL). *Porphyromonas gingivalis* (*P.gingivalis*) (3.92 log CFU/mL), *Tannerella forsythia* (*T. forsythia*) (2.98 log CFU/mL), *Treponema denticola* (*T. denticola*) (2.78 log CFU/mL), and *Prevotella intermedia* (*P. intermedia*) (2.80 log CFU/mL) exhibited moderate counts, while *Aggregatibacter actinomycetemcomitans* (*A. acitnomycetemcomitans*) (1.31 log CFU/mL) displayed the lowest counts.

**Figure 2:**
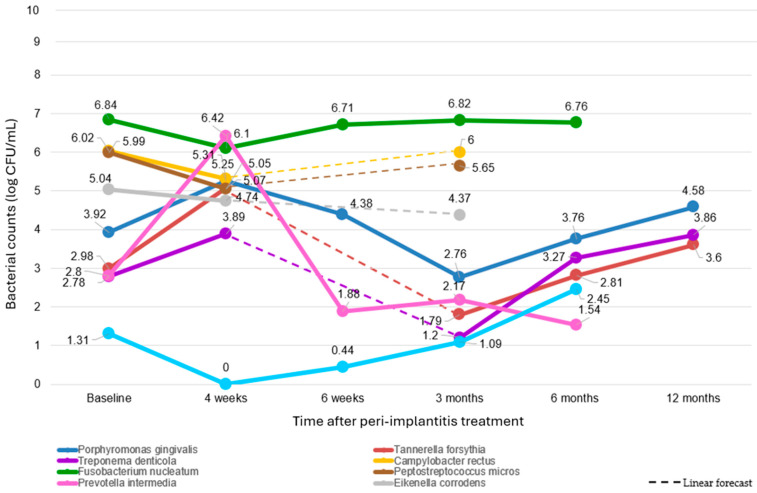
Peri-implantitis-associated microbiota variations over time before and after peri-implantitis treatment; bacterial counts (log CFU/mL) recorded at the baseline and at follow-up at 4–6 weeks and 3–6–12 months.

The extracted data showed distinct trends for each predominant pathogen analyzed in the RCTs after peri-implantitis treatment. The graph, shown in Figure 2, provides a comprehensive overview of the bacterial dynamics in treated peri-implantitis sites compared to the baseline (related data are available in Supplementary File S1).

*C. rectus* decreased to 5.31 log CFU/mL at four weeks (22 treated peri-implantitis sites [33]). At 6 weeks, data were not available. By three months, the count was 6.00 log CFU/mL (22 treated peri-implantitis sites [33]), but by 6 and 12 months, data were not available.

*P. micros* count decreased slightly to 5.05 log CFU/mL at four weeks follow-up (22 treated peri-implantitis sites [33]). At six weeks, the count was not available. By three months, the count had decreased to 5.65 log CFU/mL (22 treated peri-implantitis sites [33]). No data were available at six and twelve months.

For *T. forsythia* (22 treated peri-implantitis sites [33]), the count rose sharply to 5.07 log CFU/mL at 4 weeks after treatment. At 6 weeks, no data were available. By 3 months, the count had decreased to 1.79 log CFU/mL (62 treated peri-implantitis sites [33,35]), and at 6 months, it increased to 2.81 log CFU/mL (40 treated peri-implantitis sites [30]). The count further increased to 3.60 log CFU/mL by 12 months (40 treated peri-implantitis sites [30]).

*E. corrodens* decreased to 4.74 log CFU/mL at one month follow-up (22 treated peri-implantitis sites [33]). By three months, the count was 4.37 log CFU/mL (22 treated peri-implantitis sites [33]. No data were available at six weeks, six months, and twelve months.

*P. gingivalis* exhibited, after four weeks, a notable increase to 5.25 log CFU/mL (22 treated peri-implantitis sites [33]). By six weeks, the count decreased slightly to 4.38 log CFU/mL (19 treated peri-implantitis sites [34]). At 3 months, the count further dropped to 2.76 log CFU/mL (81 treated peri-implantitis sites [33,34,35]), but by six months, it increased again to 3.76 log CFU/mL (59 treated peri-implantitis sites [30,34]). At the 12-month mark, the count rose significantly to 4.58 log CFU/mL (40 treated peri-implantitis sites [30]).

*T. denticola* increased to 3.89 log CFU/mL at four weeks (22 treated peri-implantitis sites [33]). At six weeks no data were available. By three months, it further decreased to 1.20 log CFU/mL (62 treated peri-implantitis sites [33,35]), then increased to 3.27 log CFU/mL at six months (40 treated peri-implantitis sites [30]). At 12 months, the count was 3.86 log CFU/mL (40 treated peri-implantitis sites [30]).

*P. intermedia* increased markedly to 6.43 log CFU/mL at four weeks (22 treated peri-implantitis sites [33]). By six weeks, the count had decreased to 1.88 log CFU/mL (19 treated peri-implantitis sites [34]). At three months, the count was 2.17 log CFU/mL (81 treated peri-implantitis sites [33,34,35]) and further decreased to 1.54 log CFU/mL at six months (19 treated peri-implantitis sites [34]). No data were available at 12 months.

*A. actinomycetemcomitans* count dropped to 0.00 log CFU/mL, one month after treatment (22 treated peri-implantitis sites [33]). At six weeks, this increased to 0.44 log CFU/mL (19 treated peri-implantitis sites [34]). At 3 months, the count slightly increased to 1.09 log CFU/mL (81 treated peri-implantitis sites [33,34,35]) and further increased to 2.45 log CFU/mL at six months (19 treated peri-implantitis sites [34]). No data were available at 12 months.

*F. nucleatum* remained almost unchanged, amounting to 6.10 log CFU/mL at the one-month follow-up (22 treated peri-implantitis sites [33]), and 6.71 log CFU/mL at 6 weeks (19 treated peri-implantitis sites [34]). The count remained high at 3 months with 6.82 log CFU/mL (41 treated peri-implantitis sites [33,34]) and slightly decreased to 6.76 log CFU/mL at 6 months (19 treated peri-implantitis sites [34]). No data were available at 12 months.

### 3.4. Concomitant Trend Variations in Clinical Peri-Implant Parameters over Time After Peri-Implantitis Treatment

Clinical peri-implant data were obtained from 164 subjects involved in the six RCTs included [30,31,32,33,34,35] with 186 treated peri-implantitis sites.

If any subjects had more than one implant with peri-implantitis, three studies [31,33,34] collected the microbiological and clinical peri-implant parameters of one dental implant affected by peri-implantitis that had a higher PD. The remaining three studies [30,32,35] treated and collected the microbiological and clinical peri-implant parameters of all peri-implantitis sites treated.

#### 3.4.1. Plaque Index (PI), Modified Plaque Index (mPI/mPII), and Full Mouth Plaque Score (FMPS)

The Plaque Index (PI) [37] was recorded by assigning a dichotomous value to the absence (0) or presence (1) of plaque to calculate the percentage of treated peri-implantitis sites with the presence of plaque.

Three RCTs [30,33,34] recorded the mean percentage of PI at the implant-level, measuring the clinical parameters at six peri-implant sites (distobuccal, disto-lingual/palatal, mesiobuccal, mid-buccal, mid-lingual/palatal, and mesio-lingual/palatal) [30,32,33,34].

At baseline, 37.17% of peri-implant sites had biofilm accumulation [30,33,34] (81 treated peri-implantitis sites analyzed), and the PI dropped considerably to 13.65% at four weeks (at 22 treated peri-implantitis sites) [33]. No data were available at 6 weeks. However, it increased to 22.87% at three months (at 41 treated peri-implantitis sites [33,34]). After six months, the PI notably dropped to 15.7% (at 59 treated peri-implantitis sites [30,34]). By twelve months, the PI increased to 26.35% (at 40 treated peri-implantitis sites [30]).

The modified Plaque Index (mPI [35]/mPII [38]) described by Mombelli et al. [38] was recorded in one study [35] to assess the peri-implant plaque accumulation at four peri-implant sites (distobuccal, disto-lingual/palatal, mesiobuccal, mesio-lingual/palatal), using a graduated scale: “Score 0: no detection of plaque; score 1: plaque only recognized by running a probe across the smooth marginal surface of the implant. Implants covered by titanium spray in this area always score 1; score 2: plaque can be seen by the naked eye; score 3: abundance of soft matter” [38].

One RCT [35] (40 treated peri-implantitis sites analyzed) recorded the mean score of mPI at the baseline and after 3 months of follow-ups. The mPI mean score at the baseline was 1.13 and decreased to 0.30 at 3 months after treatment [35].

The Full Mouth Plaque Score (FMPS) was developed as a simple tool for recording soft plaque accumulation on the tooth/implant surfaces [28,39]. After all teeth/implants were examined and scored, the FMPS was obtained by dividing the number of surfaces in which there was an accumulation of plaque by the total number of surfaces examined [28,39].

Two RCTs [33,34] recorded FMPS at the baseline at 41 of the treated peri-implantitis sites analyzed, one RCT [33] recorded FMPS after 4 weeks (22 treated peri-implantitis sites analyzed), and two RCTs [33,34] recorded FMPS after 3 months (41 treated peri-implantitis sites analyzed). Baseline FMPS was 37.02%, recorded at 41 treated peri-implantitis sites [33,34], and 33.5% (at 22 treated peri-implantitis sites analyzed [33]) was recorded at one-month follow-up; six weeks after treatment, FMPS was measured at 24.47% at 19 of the treated peri-implantitis sites analyzed [34], and at three months, it was 25.88% at 41 of the treated peri-implantitis sites analyzed [33,34]. Six months post-treatment, FMPS was 20.53% in 19 subjects [34]. No data were available at 12 months.

**Figure 3:**
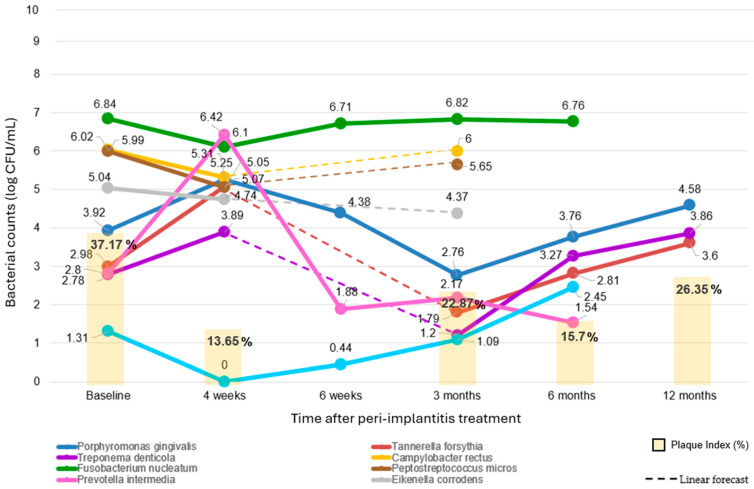
Peri-implantitis-associated microbiota and Plaque Index (%) variations after treatment over time: PI recorded at the beginning of treatment (baseline) and 1, 3, 6, and 12 months after treatment.

Concomitant trend variations in both peri-implantitis-associated microbiota and Plaque Index after peri-implantitis treatment over time are illustrated in Figure 3.

#### 3.4.2. Bleeding on Probing (BoP), Papilla Bleeding Index (PBI), Modified Sulcus Bleeding Index (mSBI), and Full Mouth Bleeding Score (FMBS)

The Bleeding on Probing (BoP) mean values were recorded using a dichotomous scale in two studies [31,32]: score 0—absence of bleeding on gentle probing; score 1—bleeding on gentle probing [40]. The BoP was recorded as a percentage of treated peri-implantitis sites bleeding on probing [40] in three studies [30,33,34].

The BoP was measured at six peri-implant sites (distobuccal, disto-lingual/palatal, mesiobuccal, mid-buccal, mesio-lingual/palatal, and mid-lingual/palatal) in four studies [30,32,33,34] and at four peri-implant sites (distobuccal, disto-lingual/palatal, mesiobuccal, and mesio-lingual/palatal) in one study [31]. Since the two studies [31,32] that recorded BoP in dichotomous form also reported the number of sites that were probed for each peri-implant site treated, it was possible to collect all the BoP values registered in all of the five included studies [30,31,32,33,34] as percentages of sites bleeding on probing. Initial BoP (68.73% at 146 peri-implantitis sites analyzed at the baseline [30,31,32,33,34]), increased to 72.51% at 4 weeks (at 22 treated peri-implantitis sites [33]). By 3 months, BoP decreased to 43.99% (at 106 treated peri-implantitis sites [31,32,33,34]). Six months post-treatment, BoP decreased to 36.66% (at 124 treated peri-implantitis sites [30,31,32,34]), and by 12 months, BoP further decreased to 22.15% (at 40 treated peri-implantitis sites [30]).

The Papilla Bleeding Index (PBI), developed by Saxer et al. [36] and Mühlemann et al. [41], was utilized in one study [35] to assess the peri-implant papilla bleeding at four peri-implant sites (distobuccal, disto-lingual/palatal, mesiobuccal, and mesio-lingual/palatal), using a graduated scale: “Score 0: no bleeding; score I A single discreet bleeding point appears; score 2: Several isolated bleeding points or a single fine line of blood appears; score 3: the interdental triangle fills with blood shortly after probing; score 4: profuse bleeding occurs after probing; blood flows immediately into the marginal sulcus” [42].

One study [35] (40 treated peri-implantitis sites analyzed) registered a mean score of PBI, measured at baseline and 3 months after peri-implantitis treatment. The PBI mean value was 1.93 at baseline and decreased to 0.43 at 3 months after treatment [35].

The modified Sulcus Bleeding Index (mSBI) described by Mombelli et al. [38] was recorded in one study [34] to assess the bleeding tendency of the peri-implant sulcus at six peri-implant sites (distobuccal, disto-lingual/palatal, mesiobuccal, mid-buccal, mesio-lingual/palatal, and mid-lingual/palatal) using a graduated scale: “Score 0: no bleeding when a periodontal probe is passed along the gingival margin adjacent to the implant; score 1: isolated bleeding spots visible; score 2: blood forms a confluent red line on margin; score 3: heavy or profuse bleeding” [38].

One RCT [34] (19 treated peri-implantitis sites analyzed) recorded a mean score of mSBI at the baseline and at 3- and 6-month follow-up. The mSBI mean score at the baseline was 1.94 and decreased to 1.01 at 3 months after treatment [34]. Six months post-treatment, the mSBI decreased to 0.98 at peri-implantitis sites [34]. No data were available after 12 months.

The Full Mouth Bleeding Score (FMBS) was developed as a simple tool for recording the tendency of bleeding during the probing of the tooth/implant surfaces [28,43]. After all teeth/implants were examined and scored, the FMBS was obtained by dividing the number of surfaces in which there was bleeding on probing by the total number of surfaces examined [28,43].

Baseline FMBS was 39.07%, recorded at 41 treated peri-implantitis sites [33,34], and FMBS was 38% (at 22 treated peri-implantitis sites analyzed [33]) at one month follow-up; no data were available at 6 weeks, but FMPS was 27.68% at three months (at 41 treated peri-implantitis sites analyzed [33,34]). Six months post-treatment, FMPS was 16.53% in 19 subjects [34]. No data were available one year after treatment.

**Figure 4:**
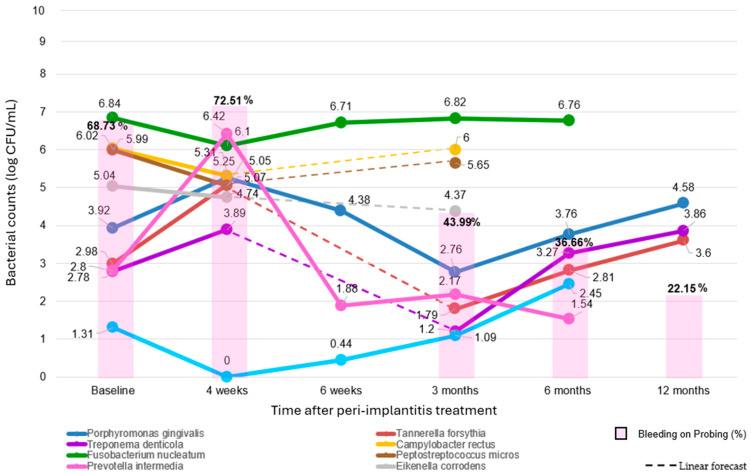
Peri-implantitis-associated microbiota and Bleeding on Probing (%) variations after treatment over time; Bleeding on Probing (BoP) was recorded at the beginning of treatment (baseline) and at 1, 3, 6, and 12 months after treatment.

Concomitant trend variations in both peri-implantitis-associated microbiota and Bleeding on Probing after peri-implantitis treatment time are illustrated in Figure 4.

#### 3.4.3. Suppuration on Probing (SoP)

The Suppuration on Probing (SoP) values [44], reported as inflammatory exudation by one included study [31], were recorded using a dichotomous scale: score 0—absence of suppuration on a gentle probing; score 1—suppuration on gentle probing [44].

One study [31] (40 treated peri-implantitis sites) registered the mean score of SoP, measured at four peri-implant sites (distobuccal, disto-lingual/palatal, mesiobuccal, and mesio-lingual/palatal), at baseline and at 3 and 6 months after peri-implantitis treatment. The SoP mean value was 0.65 at baseline and decreased to 0.05 at 3 months after treatment [5]. Six months post-treatment, the SoP further decreased to 0.15 at peri-implantitis sites [5].

#### 3.4.4. Probing Depth (PD)

Probing Depth (PD) values were recorded as the linear measurement from the mucosal margin to the pocket base, measuring the clinical parameters at six peri-implant sites (distobuccal, disto-lingual/palatal, mesiobuccal, mid-buccal, mesio-lingual/palatal, and mid-lingual/palatal) in four studies [30,32,33,34] and at four peri-implant sites (distobuccal, disto-lingual/palatal, mesiobuccal, and mesio-lingual/palatal) in two studies [31,35].

The PD mean values at the baseline were 4.98 mm (186 treated peri-implantitis sites analyzed [30,31,32,33,34,35]) and decreased to 4.6 mm at four weeks (22 treated peri-implantitis sites [33]). No study reported PD values at six weeks. By three months, the PD further decreased to 4.11 mm (146 treated peri-implantitis sites [31,32,33,34,35]). After six months, the PD improved, reaching 4.53 mm (124 treated peri-implantitis sites + [30,31,32,34]), and by twelve months, improved further to 3.95 mm (40 treated peri-implantitis sites [30]).

**Figure 5:**
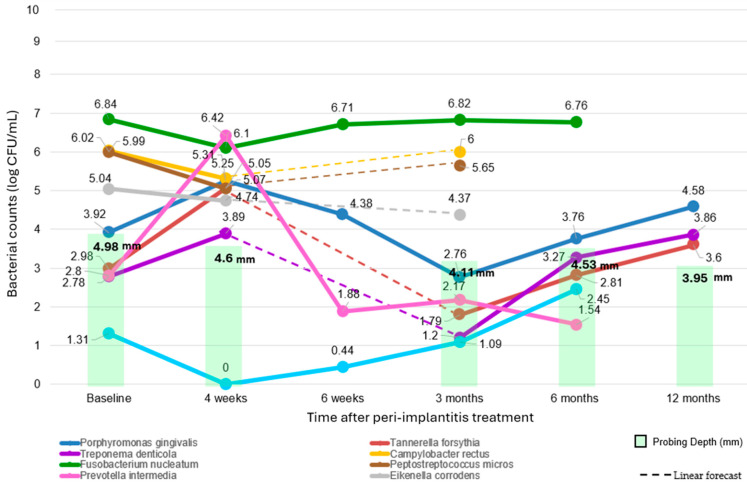
Peri-implantitis-associated microbiota and Probing Depth variations after treatment over time; Probing Depth (PD) recorded at the beginning of treatment (baseline) and 1, 3, 6, and 12 months after treatment.

Concomitant trend variations in both peri-implantitis-associated microbiota and Probing Depth after peri-implantitis treatment time are illustrated in Figure 5.

#### 3.4.5. Clinical Attachment Level (CAL)

The Clinical Attachment Level (CAL) values were recorded as the linear measurement from the cement–enamel junction to the pocket base [28].

One study [31] (40 treated peri-implantitis sites analyzed) registered the mean score of CAL, measured at four peri-implant sites (distobuccal, disto-lingual/palatal, mesiobuccal, mesio-lingual/palatal), at baseline and at 3 and 6 months after peri-implantitis treatment. The CAL mean value was −7.08 mm at baseline and decreased to −7.00 mm at 3 months after treatment [31]. Six months post-treatment, the CAL values further decreased to −6.76 mm at peri-implantitis sites [31].

### 3.5. Concomitant Trend Variations in Radiographic Peri-Implant Parameters over Time After Peri-Implantitis Treatment

One study [32] assessed changes in bone levels around peri-implantitis-treated sites (n = 23) over time, utilizing Cone Beam Computed Tomography (CBCT) to evaluate the extent of peri-implant bone loss. Marginal bone loss was defined as the linear distance from the most distal and mesial points of the implant platform to the crestal bone [45,46]. Bone level changes ≥2 mm at any time point was considered as pathological [47].

The mean deviation from baseline at the 3-month follow-up registered was 0.00 ± 2.25 (from −3.10 ± 1.64 at baseline to −3.10 ± 1.54 at 3 months) [32]. No significant differences in bone level changes were reported by the authors after 3 months at treated peri-implantitis sites [32].

The mean deviation from baseline at 6-month follow-up was −0.03 ± 2.30 (−3.10 ± 1.64 at baseline; −3.13 ± 1.61 at 6 months) [32]. No significant differences in bone level changes were reported by the authors after 6 months at treated peri-implantitis sites [32].

### 3.6. Concomitant Trend Variations in Crevicular Peri-Implant Parameters over Time After Peri-Implantitis Treatment

No data concerning the crevicular peri-implant parameters were found in any RCTs included in the present systematic review.

### 3.7. Quality Assessment

**Figure 6:**
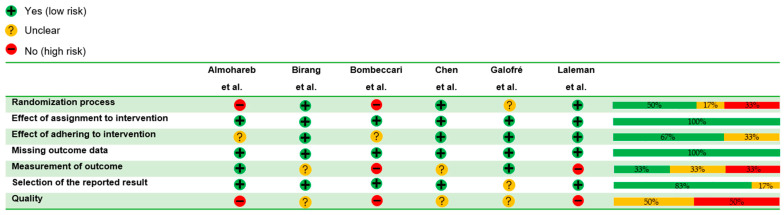
Summary and graph presented as percentages of assessment (low risk, unclear, and high risk) of the six risk of bias domains and quality judgment for the six RCTs included [30,31,32,33,34,35].

The risk of bias and the quality assessment of the six RCTs included are shown in Table 3 and the risk-of-bias graph (Figure 6). Three studies were judged as having an unclear risk of bias [32,33,35] and the other three studies as having a high risk [30,31,34].

## 4. Discussion

The present systematic review evaluated the potential impact of peri-implantitis-associated microbiota variations (total biofilm microbial load and predominant pathogen counts) on clinical peri-implant parameters recorded at 186 peri-implantitis sites after (any) treatment in 164 partially edentulous, systemically healthy, non-smoking adults from six RCTs. The limited number of included studies likely stems from restrictive eligibility criteria, which required concurrent recording of clinical (beyond radiographic and crevicular) peri-implant parameters and microbiological analysis at each follow-up visit. Indeed, no restrictions on follow-up frequency, given the nature of data, their scarcity at each time point, and heterogeneity in methodology and timing of microbiologic and clinical data collection, precluded the possibility of conducting a meta-analysis.

Furthermore, the quality assessment revealed that three studies [32,33,35] were judged as having an unclear risk of bias, and three studies [30,31,34] were judged as being high risk, primarily due to the randomization process and the measurement of the outcome. These limitations may have influenced the results and should be considered in the interpretation of the following findings.

### 4.1. Peri-Implantitis-Associated Microbiota Variations over Time After Peri-Implantitis Treatment

Data collected on microbial variation were limited and aggregated, showing considerable variability in methodology and follow-up intervals across different studies. To address this, we employed weighted averages normalizing microbial data to the number of sites sampled at each time point in any study in order to minimize bias and provide a robust comparative analysis across studies with differing sample sizes and temporal structures. Prior to peri-implantitis treatments, *F. nucleatum* exhibited the highest levels, followed by *C. rectus*, *P. micros*, *E. corrodens*, *P. gingivalis*, *T. forsythia*, *P. intermedia*, and *T. denticola*. Similarly, Van Winkelhoff et al. [48] identified *F. nucleatum*, *P. micros*, and *P. intermedia* in the majority of submucosal biofilms shortly after implant placement, suggesting that these bacteria may act as early colonizers and resident species in both healthy and diseased peri-implant niches. Additionally, peri-implantitis sites are characterized by a less diverse microbiome dominated by fewer species, notably *P. gingivalis*, *T. forsythia*, and *T. denticola*, whereas healthy implants host a more diverse and variable microbial community. The pivotal role of *F. nucleatum* in the transition from mucositis to peri-implantitis highlights its early involvement in disease progression [49].

The bacterial community in peri-implantitis undergoes interdependent changes influenced by the dynamic interactions within the biofilm. Peri-implantitis treatments reduce the overall microbial load but do not fully eradicate the dominant pathogens or other bacterial species, which exhibit heterogeneous patterns of recovery following intervention (Figure 2).

*P. gingivalis* had virulence factors that facilitate epithelial cell invasion and immune system evasion [50]. Initially, it decreased post-treatment but after displayed a variable pattern, indicating its resilience and adaptability. *T. forsythia*, a key pathogen in severe periodontitis frequently identified in peri-implantitis, exhibited a similar trend. Its persistence likely underscores its significant contribution to the disease’s pathogenic profile [51]. This bacterium’s survival tactics appeared to be linked to environmental changes and inter-species interactions. *T. denticola* showed a reduction post-treatment and subsequent variability, suggesting initial sensitivity and the ability to adapt and persist over time. This spirochete may contribute to the peri-implantitis severity by degrading tissues and inducing inflammation [52].

*P. intermedia* is detectable in healthy and peri-implantitis sites, suggesting its role as an early colonizer and adaptability [53]. Accordingly, it exhibited dramatic fluctuation, potentially indicating high reactivity to treatment and competitive pressures. This shows potential rapid overgrowth and subsequent correction.

*A. actinomycetemcomitans* experienced a dramatic decline post-treatment but gradually found a niche and began to re-establish itself, albeit at lower levels than the predominant species. Accordingly, a previous study indicated an association between *A. actinomycetemcomitans* and peri-implantitis, but not statistically significant [54], contrasting with its established role in aggressive periodontitis [55]. Its delayed but steady recovery suggests it might be outcompeted initially but can persist and increase over time as the microbial community stabilizes, highlighting the complex inter-species interactions and competitive dynamics of the microbial landscape in treated peri-implantitis sites.

Noteworthy, *F. nucleatum* maintained higher counts and appeared almost unresponsive to treatment, showing limited (or any) influence from variations in both intra-biofilm microbial changes and clinical parameters. Its sustained prevalence after treatment may be attributed to its ability to invade peri-implant tissues, swiftly recolonize treated areas, and resist common peri-implantitis treatments [56]. Similar high counts of *F. nucleatum* are typically observed before treatment at peri-implantitis, as well as at periodontitis sites [57]. It plays a crucial role in biofilm formation by acting as a bridge between early and anaerobic secondary colonizers [56], invading epithelial and endothelial cells [57], evading the immune system, surviving in different environments [58,59], and resisting antibiotics [58]. However, NSMD plus probiotics, such as *Lactobacillus reuteri*, significantly lowered *F. nucleatum* at peri-implantitis sites [60], while coaggregation with *Bifidobacterium* was effective at periodontal sites on gingival biofilms [61]. Some authors suggested the use of probiotics as adjunctive therapy in peri-implantitis treatment based on the microbial and immune–inflammatory response of the host characterizing the peri-implantitis diseases, as well as the probiotics’ role in biofilm control, host modulation, and dysbiosis reversal [17,34,62,63]. Nevertheless, the clinical practice guidelines of the European Federation of Periodontology (EFP) suggest not using probiotics as an adjunct in NSMD peri-implantitis treatment as the current evidence shows contradictory results [64]. Therefore, further studies should assess probiotics’ effects on peri-implantitis biofilm control, dysbiosis reversal, and host modulation [17].

### 4.2. Concomitant Trend Variations in Clinical Peri-Implant Parameters over Time After Peri-Implantitis Treatment

The clinical parameters valued in the six RCTs included were heterogeneous in terms of timing, methodology (e.g., 4-point vs. 6-point measurements for implants), and indices. To summarize the findings, these parameters were “normalized” across studies. No statistical significance differences and no correlation analysis in microbial or clinical results at each follow-up time could be conducted. The microbiological and clinical findings observed at the 6- to 12-month follow-ups may have been influenced by additional treatment and/or reinforcement sessions administered during the study period. It is important to note that the 12-month data are derived from one study, which may limit the generalizability of these findings. Of the six RCTs included, one described a surgical approach to peri-implantitis mechanical debridement with various adjuvant therapies. However, it was not possible to perform a comparative analysis to determine which treatment was most effective in reducing the total microbial biofilm load or the predominant pathogens counts.

The peri-implantitis sites exhibited heterogeneous severity, yet none of the proposed classifications of peri-implantitis, such as those by Monje et al. [65], Khan et al. [66], or Rosen et al. [67], were employed in any of the included RCTs.

At baseline, significant levels of many pathogenic bacteria were found, alongside elevated clinical parameters. Clinically, the Plaque Index (PI) was 37.17 ± 4.76, Bleeding on Probing (BoP) 68.73 ± 3.17, and Pocket Depth (PD) 4.98 ± 0.40 (Figure 3, Figure 5 and Figure 6), underscoring the initial peri-implantitis severity in the study population (Figure 3, Figure 5 and Figure 6).

The concomitant trend variations in PI over time after peri-implantitis treatment, assessed during follow-ups at 1 (on 22 sites) [33], 3 (41 sites) [33,34], 6 (59 sites) [30,34], and 12 (40 sites) [30] months, and generally revealed an initial drastic reduction and sustained improvement in PI, highlighting the efficacy of both surgical and non-surgical debridement [30,31,32,33,34,35]. Thus, the PI fluctuations at these time points emphasize the necessity of continuous patient management and follow-up to maintain therapeutic gains (Figure 3).

The concomitant trend variations in BoP over time after peri-implantitis treatment were analyzed at follow-ups at 1 (on 22 sites) [33], 3 (106 sites) [33], 6 (124 sites) [30,31,32,34], and 12 (40 sites) [30] months (Figure 4). The initial increase in BoP suggests a complex interaction between microbial shifts and tissue response, while the subsequent decreases and the significant long-term (12-month follow-up) reduction highlight the importance of continuous patient education, maintenance, and possible reinforcement sessions.

The concomitant trend variations in PD over time after peri-implantitis treatment were evaluated at follow-ups at 1 (on 22 sites) [33], 3 (146 sites) [31,32,33,34,35], 6 (124 sites) [30,31,32,34], and 12 (40 sites) [30] months, demonstrate lasting improvement in PD after the 3-month follow-up (Figure 5). Indeed, remarkable clinical improvements are not typically expected within the first month in the absence of more invasive treatments such as resective or regenerative procedures [68,69]. In addition, the healing process for peri-implant tissues is generally slower compared to periodontal tissues, which might explain the modest PD reduction observed at the early stage [68,69]. It is worth mentioning that, regardless of the disease classifications proposed [65,66,67] not impacting present data, the peri-implantitis case definition applied in the included RCTs is currently accepted [47]. The PD baseline and subsequent values may seem low since they are expressed as means. Notably, however, the authors of the original studies did not record any information regarding tissue/bone-level implant positioning. This lack of detailed reports on implant positioning and the overall limited (albeit expressed as mean values) variation over time may influence results interpretation and their applicability to specific clinical scenarios.

#### 4.2.1. Potential Impact of Microbial Variations After Peri-Implantitis Treatment on PI

Initially, at the 1-month mark, the PI showed a substantial reduction from a baseline value of 37.02% to 13.65% (∆ = −20.52 ± 4.76), indicating that mechanical debridement, whether surgical or non-surgical, is highly effective in reducing the overall microbial load [30,31,32,33,34,35]. However, it does not necessarily lower the predominant species. Indeed, *A. actinomycetemcomitans* was absent, and there were significant decreases in *P. micros* (5.05 ± 2.21 log CFU/mL), *C. rectus* (5.31 ± 1.63), and *E. corrodens* (4.74 ± 1.77), while *P. gingivalis* (5.25 ± 2.27), *T. forsythia* (5.07 ± 1.72), *T. denticola* (3.89 ± 3.19), and particularly *P. intermedia* (6.43 ± 1.54) interestingly increased. This phenomenon can be attributed to the loss of the “competitive balancing effect” exerted by the overall peri-implant biofilm [70]. It is plausible, in fact, that the peri-implant biofilm functions as a protective, metabolically active, and dynamically organized microbial community that competes with and balances predominant pathogens. Consequently, the quantitative (and qualitative) reduction in the peri-implant biofilm may have allowed red-complex bacteria and *P. intermedia*—which possess greater virulence, the ability to invade host cells, and obligate anaerobic metabolism—to recolonize the peri-implant niche more quickly at the expense of other bacteria.

By the 3-month follow-up, PI slightly increased to 22.87%. Although this was higher than at the 1-month follow-up, it still represented a remarkable improvement from the baseline (∆ = −14.30 ± 6.82). Notably, compared to baseline, there were increases in *P. micros* (5.65 ± 1.32 log CFU/mL), *C. rectus* (6.00 ± 0.67), and *A. actinomycetemcomitans* (1.09 ± 0.45). Conversely, *P. gingivalis* (2.76 ± 0.93), *T. forsythia* (1.79 ± 0.62), *T. denticola* (1.20 ± 0.54), *P. intermedia* (2.17 ± 0.67), *E. corrodens* (4.37 ± 1.60), and total anaerobes (4.58 ± 0.63) notably decreased. Therefore, the slight increase in PI, while still improved from baseline, may reflect a recolonization of the peri-implant environment by less pathogenic bacteria. These species, although not investigated in the RCTs included, may be more similar to those typically found in healthy peri-implant sites, predominantly colonized by *Streptococcus*, *Rothia*, *Neisseria*, and *Corynebacterium* species, along with other Gram-positive cocci and facultatively anaerobic rods [71]. These bacteria form a stable community that prevents or counteracts pathogenic colonization and maintains microbial balance [38]. It is established, in fact, that the stability and resilience of the microbial community in healthy peri-implant sites play a crucial role in maintaining peri-implant health, and that the presence of health-associated bacteria (*Streptococcus* and *Actinomyces*) is essential for preventing the pathogenic species overgrowth, thereby supporting tissue health and preventing inflammation [38]. In addition, the concurrent reduction in predominant peri-implant pathogens likely contributed to the observed improvement in PI and BoP. This demonstrates the importance of microbial dysbiosis beyond just total microbial load in peri-implant diseases [72].

At 6 months, PI further decreased to 15.70%, which could be attributed to patient education, reinforcement sessions, or additional operative sessions (10 peri-implantitis sites) [35]. Conversely, compared to 3 months, an increase in *P. gingivalis* (3.76 ± 0.66 log CFU/mL), *T. forsythia* (2.81 ± 0.85), and *T. denticola* (3.27 ± 0.69) suggests a resurgence of these pathogens. In addition, the leveling off near baseline may indicate, despite the initial improvement and the marked drop in *P. intermedia* (1.54 ± 1.53), the need for continued patient education and reinforcement sessions to maintain microbial and, especially, dysbiosis control, also considering that the total anaerobic count was recorded at 4.53 ± 0.69 (compared to baseline/3-month follow-up = 5.51. ± 0.71/4.58 ± 0.63 log CFU/mL).

By the 12-month follow-up, the PI increased to 26.35%. Despite this rise, it remained lower than baseline, indicating that while some relapse occurred, as expected [73], the overall treatment effect was still beneficial. Compared to baseline and the 3-month follow-up, *P. gingivalis* (4.58 ± 3.24 log CFU/mL), *T. forsythia* (3.60 ± 1.82), and *T. denticola* (3.86 ± 1.84) showed an increase. Additionally, the total anaerobes were consistently recorded at 4.58 ± 3.24 log CFU/mL, while the total microbial count decreased significantly to 3.60 ± 1.82. This suggests a degree of dysbiosis relapse or patient non-compliance with maintenance protocols, underscoring the necessity for continuous maintenance to prevent further relapse.

The 12-month data, derived from one study, may limit the generalizability of these findings. This underscores the necessity for more studies to validate these outcomes and elucidate the factors influencing long-term changes in PI. Despite this limitation, other studies have demonstrated similar short- and long-term trends, indicating that certain peri-implant pathogens remain at low levels up to 12 months post-treatment. In addition, the persistence of species such as *P. gingivalis* and *Treponema* species highlight the importance of ongoing maintenance and monitoring [74,75], as patients receiving supportive therapy exhibited reduced PI and BoP compared to those without support [76].

In summary, the potential impact of microbial variations after peri-implantitis treatment on PI is evident in changes in the total peri-implant biofilm microbial load, but not in the counts of predominant species. Indeed, variations in PI values over time do not consistently mirror the timing and direction of changes in predominant pathogenic species, and the discrepancy is particularly notable at the 1-month follow-up, where the trends in PI and predominant pathogens diverge.

Considering that the data were recorded as means of the more severe peri-implantitis sites in the original studies, which may account for sites with a higher progression rate (tissue destruction exceeding expectations based on biofilm deposits), as for periodontitis [77], this observation suggests that dysbiosis, characterized by the presence of specific pathogenic species rather than total microbial load, plays a crucial role in peri-implantitis.

Furthermore, both surgical and non-surgical peri-implant mechanical debridement, even with various physical/chemical adjunctive treatments, may fail to significantly impact tissue-invasive bacteria that persist locally [78], also evidenced by the almost unchanged F. nucleatum levels post-treatment. These findings highlight the need for innovative therapies that can effectively target and manage persistent peri-implantitis pathogens.

#### 4.2.2. Potential Impact of Microbial Variations After Peri-Implantitis Treatment on BoP

At the 1-month follow-up, BoP increased from a baseline value of 68.73% to 72.51% (∆ = +3.78 ± 3.17), despite a reduction in PI. This observation suggests that while the treatment effectively reduced the total microbial load, it did not immediately alleviate tissue inflammation. The initial increase in BoP may be due to the inflammatory response triggered by the reported increase in predominant pathogens, potentially explained by the loss of the “competitive balancing effect”, resulting in microbial shifts and transient inflammatory response [70]. Accordingly, BoP-positive peri-implant sites revealed higher concentrations of inflammatory markers and bacterial metabolites, including short-chain fatty and amino acids, which originate from bacterial metabolism and contribute significantly to the peri-implantitis inflammatory environment characteristic [79], underscoring the role of microbial dysbiosis in driving inflammation and tissue destruction. Therefore, BoP, beyond being a well-established inflammation indicator and potential infection, may serve as an early warning indicator of microbial imbalance in peri-implant tissues [79].

At the 3-month follow-up, a substantial reduction in BoP (∆ = −24.74 ± 5.17) was noted, which coincided with a considerable decrease in predominant pathogens, such as *P. intermedia*, *P. gingivalis*, *T. denticola*, and *T. forsythia*. These species are known to drive inflammation [50,51,52,53], and their reduction leads to decreased inflammatory mediators and lower BoP. This period likely reflects a stabilization of the microbial community, where the balance of predominant pathogens allows for tissue healing and reduced inflammation. Coherently, this time point may be ideal for case re-evaluation, as recommended for the nonsurgical phase of therapy after 6–12 weeks [76].

By the 6-month follow-up, BoP further decreased to 36.66% (∆ = −32.07 ± 5.41), suggesting that long-term patient education, regular maintenance, reinforcement, and additional operative sessions [32] play a crucial role in maintaining low inflammation levels. This stage indicates that the initial treatment’s effects are sustainable with proper follow-up care, witnessed by the PI values still lowering compared to the 3-month follow-up, resulting in healthier peri-implant environment over time. Despite a slight increase in predominant pathogens, the sustained microbial control and the overall decrease in BoP may suggest that the microbial community remained stable, balanced, and less pathogenic. This ongoing decrease in BoP underscores the importance of continuous patient education and regular maintenance in achieving long-term improvements.

At the 12-month follow-up, BoP was markedly reduced to 22.15% (∆ = −46.58 ± 6.49), demonstrating the long-term peri-implantitis treatment efficacy in inflammation control, although these results are from one study. At this point, mean BoP values were not consistent with microbiologic measurements. However, while the predominant pathogens leveled off to baseline and PI values worsened, PD continued to improve, likely suggesting that PD may reflect and/or influence BoP improvements, indirectly influencing eu/dysbiosis in peri-implant biofilm [80]. Other studies confirm these long-term findings, reporting sustained reductions in BoP up to 12 months post-treatment, proposing that the persistence of a healthy microbiota composition is essential for long-term inflammation control and emphasizing the importance of continued patient engagement and check-ups [75,81]. Accordingly, the EFP guidelines recommend rigorous maintenance protocols and supportive care, including professional mechanical plaque removal, essential for maintaining low BoP and long-term success in managing peri-implantitis [76].

In synthesis, the potential impact of microbial variations after peri-implantitis treatment on BoP is evident in the early changes in the counts of predominant species rather than the total peri-implant biofilm microbial load.

Biofilm control and the initial shifts in the microbial community can lead to transient increases in inflammation, as evidenced by peri-implant crevicular metabolic profiles and the increased BoP observed at the one-month follow-up.

Over time, changes in BoP tend to align with the timing and direction of concomitant trends in predominant pathogenic species rather than the total microbial load, as seen at the 3-month follow-up. Thus, the trend in BoP at treated peri-implantitis sites may reflect short-term variations in counts of predominant pathogens and their influence on inflammation.

#### 4.2.3. Potential Impact of Microbial Variations After Peri-Implantitis Treatment on PD

At the one-month follow-up, mean PD values decreased slightly (to 4.60 mm from a baseline value of 4.98 mm; ∆ = −0.38 ± 0.84), as expected [68,69].

By the 3-month follow-up, PD further reduced to 4.11 mm (∆ = −0.87 ± 0.51) along with the decrease in predominant pathogens and BoP. This continued improvement suggests that the debridement, combined with patient education and maintenance protocols, effectively reduces peri-implant PD over time. The reduction in PD aligns with the reduction in BoP, indicating a decrease in inflammation and pathogenic microbial load. The improvement is likely due to the combined effects of ongoing microbial control and adherence to follow-up care, which supports tissue healing and stability.

At 6 months, PD slightly increased to 4.53 mm from the 3-month value (∆ = −0.45 ± 0.57), this may reflect variations in patient adherence to maintenance protocols or the need for any additional reinforcement sessions [82]. However, the PD remained lower than the baseline, indicating that the treatment effects may be sustained over the medium term.

By the 12-month follow-up, PD further reduced to 3.95 mm (∆ = −1.03 ± 0.51), in addition to the resurgence of the predominant pathogens counts and PI and BoP worsening. Although derived from one study, which may limit the generalizability of these findings, the sustained improvement in PD at 12 months highlights the importance of continuous patient management and maintenance to achieve and maintain favorable clinical outcomes over the long term [75,81]. Comparative studies have also shown that adjunctive treatments, such as local minocycline, can enhance PD reduction. For instance, local minocycline with surgical treatment significantly improved PD and CAL at the 6-month follow-up, suggesting its potential effect as adjunctive therapy [83]. However, the benefits of systemic antibiotics like metronidazole and amoxicillin were not significant, highlighting the importance of selecting adjunctive treatments based on the severity and peri-implantitis cases [84]. The EFP guidelines stress the importance of integrating supportive peri-implant care and appropriate adjunctive treatments to maintain reduced PD long-term. This approach aligns with our findings, emphasizing continuous patient engagement and professional maintenance to achieve optimal outcomes in peri-implant health [76].

In conclusion, the potential impact of microbial variations after peri-implantitis treatment on PD can be observed in the medium to long-term (6- and 12-month follow-ups). Over time, variations in PD tend to retrace or influence the trend of concomitant changes in predominant pathogenic species, particularly evident at the 3- and 6-month follow-ups, indicating that PD changes more slowly compared to PI and BoP.

However, rather than reflecting immediate microbial load, PD might influence the peri-implant microenvironment [80] in a way that favors the predominant pathogenic species. Therefore, PD, along with BoP, provides crucial clinical evidence for measuring treatment efficacy and determining indications for treatment and retreatment [76].

### 4.3. Strengths, Limits, and Future Perspectives

The present systematic review is possibly the first to evaluate, albeit only descriptively, concomitant trends in both microbial (total load and predominant pathogens’ counts) and clinical, radiographic, and crevicular variations following peri-implantitis treatment and, based on current evidence, to speculate on the potential impact of the peri-implantitis-associated microbiota variations on peri-implant clinical parameters (PI, BoP, PD) in partially edentulous, systemically healthy, non-smokers.

The clinical data collected were limited and aggregated, showing considerable variability in methodology and follow-up intervals across different studies. This variability complicated comparisons and necessitated conversions into more common parameters. The methodological differences also hindered a quantitative analysis of the results, making it challenging to evaluate the significance of differences in both microbial and clinical parameters at follow-ups and to assess potential correlations between them.

Future research should aim to standardize the methodology and follow-up intervals to facilitate more reliable comparisons and quantitative analyses. A consistent approach across studies would allow for a more accurate evaluation of the potential impact of different treatments on both microbial and clinical parameters.

Additionally, there were no data available on treated peri-implant mucositis sites and total edentulism. Future studies should include these parameters and the patient’s periodontal history to better understand the microbiological and the course of clinical outcomes in a broader patient population [85]. It is essential to consider that individuals with peri-implant mucositis may harbor an altered submucosal microbiota, even in healthy periodontal and peri-implant sites [69]. Moreover, since the data were obtained exclusively from dentate subjects, it is important to explore the microbial reservoirs in the crevicular and subgingival areas of natural teeth, which may play a role in recolonizing the submucosal area around dental implants in partially edentulous patients [86]. In contrast, totally edentulous subjects with dental implants might exhibit different microbial profiles due to the fundamental electrophysical differences between titanium dioxide in dental implants and hydroxyapatite in natural teeth [87]. Titanium dioxide or other metal alloys can influence biofilm composition and alter host immune responses [69]. Additionally, titanium concentrations in dental plaque are inversely correlated with species richness in healthy peri-implant sites, highlighting the potential impact of titanium particles on the peri-implant microbiome [69].

Moreover, radiographic data were not discussed due to the inability to make comparisons among follow-ups and the limited maximum duration (12 months) of outcomes recorded in the original studies. Only one study [32] included in the present systematic review reported on bone level changes around treated peri-implantitis sites (n = 23) over time after peri-implantitis treatment using CBCT images to define the extension of peri-implant bone loss as marginal bone loss. No significant differences in bone level changes were reported by the authors after 3 months at treated peri-implantitis sites [32].

Even if consensus has not been achieved regarding radiographic images being most suitable for the assessment of peri-implantitis bone defects, intraoral radiography is frequently used as a first-line imaging technique due to the lower associated radiation dose [88,89,90,91]. The use of CBCT in the assessment of peri-implant bone defects is indicated to overcome the limitations of the two-dimensional intraoral or panoramic radiography for more complex cases and if the combination of clinical and two-dimensional radiography information was insufficient [88,90]. In fact, CBCT can assist clinicians in the evaluation of the vestibular and lingual/palatal cortical bone level and in the evaluation of crater-like bone defects [88,90]. Furthermore, as geometrical distortion is inherent in two-dimensional radiography images, CBCT allows for the undistorted determination of peri-implant bone defects [88,90]. Pelekos et al. [91] reported that intraoral radiography accuracy in detected bone defects was affected by bone defect morphology, bone wall thickness, time of exposure, and radiologist experience. In contrast, the CBCT diagnostic accuracy was registered as a major 96% for all bone defect types [91]. The disadvantage of CBCT can, however, be the artifacts generated by dense materials with high anatomical number, such as implant titanium [89]. Numerous algorithms for reducing metal artifacts have been developed for CBCT, although unanimous recommendations on their use have not yet been reached [89].

Future studies should include longer follow-up periods and standardized radiographic assessments to provide a more comprehensive understanding of bone level changes over time.

Additionally, no crevicular data were obtained from the included studies, which could have provided insights into the sub-/pre-clinical peri-implant tissue response to microbial variations. Indeed, distinct metabolic signatures in the peri-implant crevicular fluid of peri-implantitis sites with elevated levels of inflammatory markers and metabolic by-products, indicative of an intensified inflammatory response and altered metabolic processes, have been described. Notably, metabolites such as short-chain fatty acids and amino acids are found in higher concentrations in peri-implantitis sites, thus reflecting the metabolic activities of the pathogenic bacteria present [79]. Incorporating crevicular fluid analysis, including peri-implant pH assessment that could play a role in bacterial composition by favoring or inhibiting different bacterial species [88,89,90], in future research, could offer valuable information on the inflammatory and immune responses associated with peri-implant diseases, and metabolic profiles.

Finally, among the included studies, NSMD peri-implantitis treatments were the most commonly performed [30,32,33,34,35], while SMD was used in only one study [31]. NSMD alone emerged as the predominant treatment type, particularly in control groups. However, significant heterogeneity in adjunctive treatments (e.g., local antibiotics, aPDT, laser, air-polishing, antimicrobial mouthwashes, and probiotics) was recorded [30,31,32,33,34,35]. It should be considered that adjunctive treatments may have determined a greater reduction in some microbiological species than others, while for clinical parameters, no adjunctive treatment was superior to the others and current evidence on their influence on different clinical parameters is still controversial [92,93]. Notably, none of the included RCTs performed resective or regenerative surgical procedures for peri-implantitis treatments. These surgical treatments, typically indicated for single-walled or horizontal bone defects, could significantly alter the peri-implantitis site anatomies, potentially influencing the microbiological load and composition more rapidly than other treatments [94,95]. Subsequent studies should evaluate the potential impact of each additional treatment for peri-implantitis on changes in the microbiota associated with peri-implantitis and correlations with clinical, radiographic, crevicular, and peri-implant parameters.

Addressing these limitations and expanding the scope of future clinical studies, with larger studies, exploring additional interventions, and recording long-term outcomes, a more thorough understanding of the microbial and clinical dynamics in peri-implantitis can be achieved, ultimately leading to more effective prevention and treatment strategies.

### 4.4. Clinical Relevance

- Mechanical debridement alone or with adjunctive treatment failed to eradicate predominant pathogens and other species from the peri-implantitis sites, which were likely to be recolonized.
- The potential impact of microbial variations after peri-implantitis treatment on PI is evident in changes in the total peri-implant biofilm microbial load, but not in the counts of predominant species. Indeed, variations in PI values over time do not consistently mirror the timing and direction of changes in predominant pathogenic species, and the discrepancy is particularly notable at the 1-month follow-up, where the trends in PI and predominant pathogens diverge.
- Considering that the data were recorded as means of the more severe and/or refractory peri-implantitis sites in the original studies, which may account for sites with a higher rate of progression (tissue destruction exceeding expectations based on biofilm deposits), as graded for periodontitis, this observation suggests that dysbiosis, characterized by the presence of specific pathogenic species rather than the total microbial load, plays a crucial role in peri-implantitis.
- Both surgical and non-surgical methods for peri-implant mechanical debridement, even when supplemented with various chemical or physical adjunctive therapies, often have limited efficacy against tissue-invasive bacterial species that remain localized [83]. This is further demonstrated by the minimal reduction in *Fusobacterium nucleatum* levels following treatment. These observations underscore the necessity for innovative therapeutic approaches capable of effectively addressing and controlling persistent pathogens in the peri-implant environment.
- The potential impact of microbial variations after peri-implantitis treatment on BoP is evident in the early changes in the counts of predominant species rather than the total peri-implant biofilm microbial load. Indeed, biofilm control and the initial shifts in the microbial community can lead to transient increases in inflammation, as evidenced by peri-implant crevicular metabolic profiles and the increased BoP observed at the one-month follow-up. However, over time, changes in BoP values tend to align with the timing and direction of concomitant trends in predominant pathogenic species rather than the total microbial load, as seen at the three-month follow-up. Thus, the trend in BoP at treated peri-implantitis sites may reflect short-term variations in counts of predominant pathogens and their influence on inflammation.
- The potential impact of microbial variations after peri-implantitis treatment on PD can be observed in the medium to long term (6- and 12-month follow-ups). Over time, variations in PD values tend to retrace or follow the trend of concomitant changes in predominant pathogenic species, particularly evident at the 3- and 6-month follow-ups, indicating that PD is a parameter that changes more slowly compared to PI and BoP.
- However, rather than reflecting immediate microbial load, PD might influence the microenvironment of the peri-implant niche [80] in a way that favors the predominant pathogenic species. Therefore, PD, along with BoP, provides crucial clinical evidence for measuring treatment efficacy and determining indications for treatment and retreatment [76].
- Targeted and timely therapeutic approaches should be explored to effectively manage the overall microbial load and persistent dominant pathogens in peri-implantitis sites.

## 5. Conclusions

Peri-implantitis treatment markedly reduced the total microbial load, although it did not eradicate predominant pathogens and other species, which showed heterogeneous recovery patterns.

PI showed an immediate substantial reduction following treatment, indicating that mechanical debridement, whether surgical or non-surgical, is highly effective in reducing the overall microbial load. However, it does not necessarily lower the counts of predominant species, likely due to the loss of the “competitive balancing effect” of the peri-implant biofilm. Consequently, virulent bacteria like red-complex species and *P. intermedia* recolonize the peri-implant niche more quickly. By the three-month follow-up, a slight increase in PI reflects recolonization by less pathogenic species and concurrent reduction in predominant pathogens. At six months, PI further decreased, then increased again by the twelve-month mark.

At the one-month follow-up, BoP slightly increased despite a reduction in PI and changes in BoP aligned with trends in predominant pathogens, where biofilm control and initial microbial shifts can lead to transient increases in inflammation. By the three-month follow-up, BoP significantly reduced, coinciding with a decrease in predominant pathogens. Further reductions at six and twelve months demonstrated the long-term efficacy of peri-implantitis treatments.

PD values decreased by the three-month follow-up, coinciding with decreases in pathogens and BoP. At six months, PD slightly increased to 4.53 mm but remained lower than baseline. Despite a resurgence in pathogen counts and worsening of PI and BoP, PD decreased again by the twelve-month follow-up. PD values tend to follow trends in predominant pathogenic species, particularly at three and six months. PD changes were somewhat comparable to PI and BoP.

Even with adjunctive treatments, both surgical and non-surgical peri-implant mechanical debridement fail to significantly impact tissue-invasive bacteria like *F. nucleatum*, which maintained high counts post-treatment and seemed unaffected by clinical or intra-microbiota variations.

## Figures and Tables

**Table 1:** RCT characteristics. Studies: first author, year and journal of publication, study design, reference, quality, and funding. Population: sample size (n.), mean age and range (y.o.), and gender ratio (M/F). Peri-implant site: total number (n.), peri-implantitis sites number (n.), implant design, type, level (soft tissue, bone), abutment (type), supported restoration (type and number), and mean time after implant placement. Peri-implantitis treatment: type, removal of the prosthesis (yes or no), and session (n.).

Studies	Population	Peri-Implant Site	Peri-Implantitis Treatment
Almohareb T., 2020 *Photodiagnosis Photodyn Ther* [30] RCT High risk Deanship of Scientific Research, King Saud University	Group 1: n. 20 Mean age: 51.7 ± 7.5 y.o. Gender ratio: 18M/2F	Peri-implant site(s): n. 43 Peri-implantitis sites: n. 20 Dental implant type and design: MD Dental implant level (soft tissue, bone): MD Abutment type: MD Supported restoration: MD (Mean) time elapsed after implant positioning: MD	NSMD + Local antibiotics (500 mg AMX for 3 days + 400 mg MTZ for 7 days) + CHX (0.12% twice a day) + Diode laser + aPDT Prosthesis removal: MD Session: n. 1
	Group 2: n. 20 Mean age: 50.9 ± 6.3 y.o. Gender ratio: 16M/4F	Peri-implant site(s): n. 36 Peri-implantitis sites: n. 20 Dental implant type and design: MD Dental implant level (soft tissue, bone): MD Abutment type: MD Supported restoration: MD (Mean) time elapsed after implant positioning: MD	NSMD + Local antibiotics (500 mg AMX for 3 days + 400 mg MTZ for 7 days) + CHX (0.12% twice a day) + Diode laser Prosthesis removal: MD Session: n. 1
Birang E., 2017 *J Laser Med Sci* [35] RCT Unclear risk No Funding	Group 1: n. 10 Mean age: N/D Gender ratio: N/D	Peri-implant site(s): MD Peri-implantitis sites: n. 20 Dental implant type and design: MD Dental implant level (soft tissue, bone): MD Abutment type: MD Supported restoration: MD (Mean) time elapsed after implant positioning: MD	NSMD + Air polishing (Prophy-Jet) + Diode laser + aPDT Prosthesis removal: MD Session: n. 2
	Group 2: n. 10 Mean age: N/D Gender ratio: N/D	Peri-implant site(s): n. MD Peri-implantitis sites: n. 20 Dental implant type and design: MD Dental implant level (soft tissue, bone): MD Abutment type: MD Supported restoration: n. MD (Mean) time elapsed after implant positioning: MD	NSMD + Air polishing (Prophy-Jet) + Diode laser Prosthesis removal: MD Session: n. 2
Bombeccari, G.P., 2013 *Implant Dent* [31] RCT High risk No Funding	Group 1: n. 20 Mean age: N/D Gender ratio: N/D	Peri-implant site(s): n. MD Peri-implantitis sites: n. 20 Dental implant type and design: Nobel Biocare^®^ with rough surfaces Dental implant level (soft tissue, bone): MD Abutment type: MD Supported restoration: n. MD (Mean) time elapsed after implant positioning: MD	SMD + Local CHX (0.2%) + Diode laser + aPDT + CHX (0.2%, 10 mL for 1 min once every 8 h for 2 weeks) Prosthesis removal: no Session: n. 1
	Group 2: n. 20 Mean age: N/D Gender ratio: N/D	Peri-implant site(s): n. MD Peri-implantitis sites: n. 20 Dental Implant type and design: Nobel Biocare^®^ with rough surface Dental Implant Level (soft tissue, bone): MD Abutment type: MD Supported restoration: n. MD (Mean) time elapsed after implant positioning: MD	SMD + Local CHX (0.2%) + CHX (0.2%, 10 mL for 1 min once every 8 h for 2 weeks) Prosthesis removal: no Session: n. 1
Chen, J.H., 2022 *Laser Med Sci* [32] RCT Unclear risk Southern Taiwan Science Park	Group 1: n. 11 Mean age: MD Gender ratio: MD	Peri-implant site(s): n. MD Peri-implantitis sites: n. 13 Dental implant type and design: MD Dental implant level (soft tissue, bone): MD Abutment type: MD Supported restoration: n. MD (Mean) time elapsed after implant positioning: MD	Er:YAG laser (SA-108, SAPPHIRE, LightMed) Prosthesis removal: MD Session: n. 3 at baseline, at 2 and 4 weeks
	Group 2: n. 12 Mean age: MD Gender ratio: MD	Peri-implant site(s): n. MD Peri-implantitis sites: n. 12 Dental implant type and design: MD Dental implant level (soft tissue, bone): MD Abutment type: MD Supported restoration: n. MD (Mean) time elapsed after implant positioning: MD	NSMD Prosthesis removal: MD Session: n. 1
Galofré, M., 2018 *J Periodontal Res* [33] RCT Unclear risk Sunstar Suisse and BioGaia	Group 1: n. 11 Mean age: 61.7 ± 7.0 Gender ratio: 8M/3F	Peri-implant site(s): n. MD Peri-implantitis sites: n. 11 Dental implant type and design: MD Dental implant level (soft tissue, bone): MD Abutment type: MD Supported restoration: single crown (n. 36); fixed partial prosthesis (n. 64) (Mean) time elapsed after implant positioning: MD	NSMD + *Lactobacillus reuteri* (Prodentis, PerioBalance^®^, 1 lozenges for 30 days) Prosthesis removal: MD Session: n. 1
	Group 2: n. 11 Mean age: 56.8 ± 9.3 Gender ratio: 5M/6F	Peri-implant site(s): n. MD Peri-implantitis sites: n. 11 Dental implant type and design: MD Dental implant level (soft tissue, bone): MD Abutment type: MD Supported restoration: single crown (n. 36); fixed partial prosthesis (n. 64) (Mean) time elapsed after implant positioning: MD	NSMD + Placebo (1 lozenges for 30 days) Prosthesis removal: MD Session: n. 1
Laleman, I., 2020 *Clin Oral Implants Res* [34] RCT High risk BioGaia AB and Acteon	Group 1: n. 9 Mean age: 64 ± 11 Gender ratio: 5M/4F	Peri-implant site(s): n. MD Peri-implantitis sites: n. 9 Dental implant type and design: N/D Dental implant level (soft tissue, bone): N/D Abutment type: N/D Supported restoration: N/D (Mean) time elapsed after implant positioning: N/D	NSMD + Powder air-polishing (Air-N-Go Easy, Acteon) + Probiotic (*Lactobaillus reuteri*, BioGaia AB) Prosthesis removal: MD Session: n. 1
	Group 2: n. 10 Mean age: 69 ± 9 Gender ratio: 4M/6F	Peri-implant site(s): n. MD Peri-implantitis sites: n. 10 Dental implant type and design: N/D Dental implant level (soft tissue, bone): N/D Abutment type: N/D Supported restoration: N/D (Mean) time elapsed after implant positioning: N/D	NSMD + Powder air-polishing (Air-N-Go Easy) + Placebo Prosthesis removal: MD Session: n. 1

Abbreviations: Randomized controlled trial, “RCT”; number, “n”; male, “M”; female, “F”; years old, “y.o.”; missing data, “MD”; not defined, “N/D”; non-surgical mechanical debridement, “NSMD”; surgical mechanical debridement, “SMD”; antimicrobial photodynamic therapy, “aPDT”; chlorhexidine, “CHX”; amoxicillin, “AMX”; metronidazole, “MTZ”; erbium-doped yttrium aluminum garnet, “Er:YAG”; percentage, “%”; milliliters, “mL”; milligrams, “mg”.

**Table 2:** Peri-implant total microbial load and predominant pathogens’ counts, at the baseline (before peri-implantitis treatment) and at follow-up at 4 weeks, 6 weeks, 3 months, 6 months, and 12 months. Studies characteristics: reference, group (1/2), treated peri-implantitis sites (n.), and peri-implantitis treatment. Peri-implant microbial load and predominant pathogens’ counts (log CFU/mL) at (treated) peri-implantitis sites: *Porphyromonas gingivalis*, *Tannarella forsythia*, *Treponema denticola*, *Fusobacterium nucleatum*, *Prevotella intermedia*, *Peptostreptococcus micros*, *Campylobacter rectus*, *Aggregatibacter actinomycetemcomitans*, *Eikenella corrodens*, total anaerobes, and total microbial load. Peri-implant clinical parameters at (treated) peri-implantitis sites: Plaque Index (%), Clinical Attachment Level (mm), modified Plaque Index (mean score), Probing Depth (mm), Bleeding on Probing (%), Papilla Bleeding Index (mean score), modified Sulcus Bleeding Index (mean score), and Suppuration on Probing (mean score). Full mouth clinical parameter scores: Full Mouth Plaque Score (%) and Full Mouth Bleeding Score (%).

Studies	Peri-Implant Microbial Load and Predominant Pathogen Counts (log CFU/mL)											Peri-Implant Clinical Parameters									
	(Treated) Peri-Implantitis Sites											(Treated) Peri-Implantitis Sites								Full Mouth Scores	
	P.g.	T.f.	T.d.	F.n.	P.i.	P.m.	C.r.	A.a.	E.c.	Total Anaerobes	Total Load	PI (%)	CAL (mm)	mPI (Mean Score)	PD (mm)	BoP (%)	PBI (Mean Score)	mSBI (Mean Score)	SoP (Mean Score)	FMPS (%)	FMBS (%)
Before peri-implantitis treatment (baseline)																					
Almohareb [30] Group 1 n. 20	5.73 ± 1.12	4.22 ± 1.73	4.19 ± 1.92									38.6 ± 9.5			5.2 ± 2.0	45.3 ± 14.8					
Group 2 n. 20	5.29 ± 1.64	4.46 ± 1.21	4.54 ± 1.08									41.2 ± 11.7			5.4 ± 2.1	43.8 ± 13.9					
Birang [35] Group 1 n. 20	1.42 ± 1.49	0.43 ± 0.55	0.53 ± 0.63		1.04 ± 1.30			0.91 ± 0.80						1.25 ± 0.64	4.06 ± 0.78		1.85 ± 0.87				
Group 2 n. 20	1.68 ± 1.50	0.31 ± 0.55	0.48 ± 0.55		1.27 ± 1.11			1.12 ± 0.86						1.01 ± 0.91	4.02 ± 0.67		2.00 ± 0.86				
Bombeccari [31] Group 1 n. 20										2.35 ± 0.02			−7.11 ± 0.02		5.90 ± 0.76	70			0.70 ± 0.48		
Group 2 n. 20										2.37 ± 0.03			−7.05 ± 0.02		5.80 ± 0.78	80			0.60 ± 0.51		
Chen [32] Group 1 n. 13										9.23 ± 3.06					4.95 ± 1.72	61.5					
Group 2 n. 12										12.02 ± 1.90					3.65 ± 1.46	58.33					
Galofrè [33] Group 1 n. 11	5.20 ± 2.90	5.46 ± 1.20	3.80 ± 3.16	6.78 ± 0.97	6.10 ± 2.34	5.88 ± 0.78	5.97 ± 1.16	0.00 ± 0.00	4.36 ± 2.94		9.05 ± 1.11	63.6			5.07 ± 0.87	100				44 ± 14	53 ± 23
Group 2 n. 11	4.81 ± 3.29	5.06 ± 1.87	4.33 ± 2.92	6.81 ± 0.66	6.43 ± 2.22	6.10 ± 0.61	6.07 ± 0.86	0.00 ± 0.00	5.72 ± 1.12		9.31 ± 0.67	45.5			4.90 ± 0.66	90.9				43 ± 21	49 ± 23
Laleman [34] Group 1 n. 9	5.13 ± 3.14			6.93 ± 0.78	2.46 ± 1.97			3.09 ± 2.54				15 ± 13			5.17 ± 0.92	87 ± 13		1.92 ± 0.70		29 ± 11	30 ± 10
Group 2 n. 10	3.51 ± 3.37			6.87 ± 0.90	2.04 ± 2.28			3.74 ± 2.47				8 ± 21			5.45 ± 1.20	87 ± 22		1.96 ± 0.79		30 ± 14	21 ± 13
X	3.92 ± 0.13	2.98 ± 1.12	2.78 ± 1.48	6.84 ± 0.42	2.80 ± 0.71	5.99 ± 0.70	6.02 ± 0.72	1.31 ± 0.52	5.04 ± 1.57	5.51. ± 0.71	9.18 ± 0.65	37.17 ± 4.76	−7.08 ± 0.01	1.13 ± 0.56	4.98 ± 0.40	68.73 ± 3.17	1.93 ± 0.61	1.94 ± 0.53	0.65 ± 0.35	37.02 ± 7.96	39.07 ± 9.54
4-week follow-up																					
Galofrè [33] Group 1 n. 11	5.74 ± 3.08	5.60 ± 1.09	4.04 ± 3.26	5.60 ± 2.92	7.18 ± 0.88	4.81 ± 2.48	4.95 ± 2.58	0.00 ± 0.00	4.48 ± 2.99		9.46 ± 0.93	9.1			4.55 ± 0.69	54.5				31 ± 12	34 ± 6
	∆: +0.54 ± 4.23	∆: + 0.14 ± 1.62	∆: + 0.24 ± 4.54	∆: −1.18 ± 3.08	∆: + 1.08 ± 3.22	∆: −1.07 ± 2.60	∆: −1.02 ± 2.83	∆: 0.00 ± 0.00	∆: + 0.12 ± 4.19		∆: +0.41 ± 1.45	∆: −54.5			∆: −0.52 ± 1.11	∆: −45.5				∆: −13 ± 18.44	∆: −19 ± 23.77
Group 2 n. 11	4.75 ± 3.34	4.54 ± 2.34	3.73 ± 3.12	6.59 ± 0.72	5.67 ± 2.96	5.30 ± 1.94	5.67 ± 1.98	0.00 ± 0.00	5.00 ± 1.88		9.26 ± 0.66	18.2			4.65 ± 0.78	90.9				36 ± 16	42 ± 22
	∆: −0.06 ± 4.69	∆: −0.52 ± 3.00	∆: −0.60 ± 4.27	∆: −0.22 ± 0.98	∆: −0.76 ± 3.70	∆: −0.80 ± 2.04	∆: −0.40 ± 2.16	∆: 0.00 ± 0.00	∆: −0.72 ± 2.19		∆: −0.05 ± 0.94	∆: −27.3			∆: −0.25 ± 1.02	∆: 0.00				∆: −7 ± 26.40	∆: −7 ± 31.83
X	5.25 ± 2.27	5.07 ± 1.72	3.89 ± 3.19	6.10 ± 2.14	6.43± 1.54	5.05 ± 2.21	5.31 ± 1.63	0.00 ± 0.00	4.74 ± 1.77		9.36 ± 0.57	13.65			4.60 ± 0.74	72.51				33.5 ± 10.00	38 ± 11.40
*D*	−1.65 ± 2.27	+2.09 ± 2.05	+1.11 ± 3.51	−0.74 ± 2.18	+ 3.63 ± 1.70	−0.94 ± 2.32	−0.71 ± 1.78	−1.31 ± 0.52	−0.30 ± 2.37		+0.18 ± 0.86	−20.52 ± 4.76			−0.38 ± 0.84	+3.78 ± 3.17				−3.52 ± 12.78	+1.07 ± 14.86
6-week follow-up																					
Laleman [34] Group 1 n. 9	5.27 ± 3.10			6.69 ± 0.94	2.41 ± 2.44			3.71 ± 1.66												25 ± 11	
	∆: +0.14 ± 4.41			∆: −0.24 ± 1.22	∆: −0.05 ± 3.14			∆: +0.62 ± 3.04												∆: −4 ± 15.56	
Group 2 n. 10	3.49 ± 3.33			6.72 ± 1.29	1.35 ± 2.26			3.67 ± 2.30												24 ± 8	
	∆: −0.02 ± 4.74			∆: −0.15 ± 1.57	∆: −0.69 ± 3.21			∆: −0.07 ± 3.38												∆: −6 ± 16.12	
X	4.38 ± 2.29			6.71 ± 0.81	1.88 ± 1.66			0.44 ± 1.44												24.47 ± 4.49	
*D*	+0.46 ± 2.29			−0.13 ± 0.91	−0.92 ± 1.81			−0.87 ± 1.53												−12.55 ± 9.14	
3-month follow-up																					
Birang [35] Group 1 n. 20	0.70 ± 0.99	0.14 ± 0.24	0.21 ± 0.46		0.39 ± 0.58			0.47 ± 0.64						0.35 ± 0.49	2.75 ± 0.84		0.50 ± 0.61				
	∆: −0.72 ± 1.79	∆: −0.29 ± 0.6	∆: −0.32 ± 0.78		∆: −0.65 ± 1.42			∆: −0.44 ± 1.02						∆: −0.90 ± 0.81	∆: −1.31 ± 1.15		∆: −1.35 ± 1.06				
Group 2 n. 20	1.03 ± 1.44	0.15 ± 0.27	0.28 ± 0.44		0.65 ± 1.19			0.61 ± 0.62						0.25 ± 0.44	2.69 ± 0.77		0.35 ± 0.59				
	∆: −0.65 ± 2.08	∆: −0.16 ± 0.61	∆: −0.20 ± 0.71		∆: −0.62 ± 1.63			∆: −0.51 ± 1.06						∆: −0.76 ± 1.01	∆: −1.33 ± 1.02		∆: −1.65 ± 1.04				
Bombeccari [31] Group 1 n. 20										1.50			−6.58 ± 0.02		5.20 ± 1.03	0.00			0.00 ± 0.00		
										∆: −0.85 ± 0.02			∆: +0.53 ± 0.02		∆: −0.70 ± 1.28	∆: −70			∆: −0.70 ± 0.00		
Group 2 n. 20										1.86			−7.02 ± 0.02		5.70 ± 0.48	30			0.10 ± 0.31		
										∆: −0.51 ± 0.03			∆: +0.03 ± 0.02		∆: −0.1 ± 0.92	∆: −50			∆: −0.50 ± 0.60		
Chen [32] Group 1 n. 13										9.43 ± 1.85					4.11 ± 2.09	57.67					
										∆: +0.20 ± 3.57					∆: −0.84 ± 2.71	∆: −3.83					
Group 2 n. 12										9.05 ± 2.74					3.15 ± 1.94	43					
										∆: −2.97 ± 3.34					∆: −0.50 ± 2.43	∆: −15.33					
Galofrè [33] n. 11	5.21 ± 2.86	4.78 ± 2.45	3.14 ± 3.14	6.64 ± 1.18	6.06 ± 2.18	5.32 ± 1.94	5.80 ± 1.02	0.00 ± 0.00	3.77 ± 2.66		8.96 ± 1.10	36.4			4.53 ± 0.72	63.6				28 ± 24	33 ± 9
	∆: +0.01 ± 4.07	∆: −0.68 ± 2.73	∆: −0.66 ± 4.45	∆: −0.14 ± 1.53	∆: −0.04 ± 4.52	∆: −0.56 ± 2.09	∆: −0.17 ± 1.54	∆: 0.00 ± 0.00	∆: −0.59 ± 3.96		∆: −0.09 ± 1.56	∆: −27.2			∆: −0.54 ± 1.13	∆: −36.4				∆: −16 ± 27.78	∆: −20 ± 24.70
Group 2 n. 11	4.91 ± 3.43	4.89 ± 2.48	3.30 ± 3.26	6.94 ± 0.50	5.47 ± 2.91	5.97 ± 0.69	6.20 ± 0.87	0.00 ± 0.00	4.96 ± 1.79		9.33 ± 0.74	36.4			4.70 ± 0.75	90.9				33 ± 28	39 ± 17
	∆: +0.10 ± 4.75	∆: −0.17 ± 3.11	∆: −1.03 ± 4.37	∆: + 0.13 ± 0.83	∆: −0.96 ± 3.66	∆: −0.13 ± 0.92	∆: + 0.13 ± 1.73	∆: 0.00 ± 0.00	∆: − 0.76 ± 2.11		∆: +0.02 ± 1.00	∆: −9.1			∆: −0.20 ± 1.00	∆: 0.00				∆: −10 ± 35.00	∆: −10 ± 28.60
Laleman [34] Group 1 n. 9	5.22 ± 3.16			6.84 ± 1.21	1.53 ± 2.39			3.62 ± 2.43				3 ± 7			4.13 ± 1.04	63 ± 31		1.14 ± 0.88		20 ± 11	19 ± 10
	∆: +0.09 ± 4.45			∆: −0.09 ± 1.44	∆: −0.93 ± 3.10			∆: + 0.53 ± 3.51				∆: −12 ± 14.76			∆: −1.04 ± 1.39	∆: −24 ± 33.61		∆: −0.78 ± 1.13		∆: −9 ± 15.56	∆: −11 ± 14.14
Group 2 n. 10	3.08 ± 3.48			6.87 ± 1.21	1.40 ± 2.32			3.43 ± 2.33				11 ± 19			4.30 ± 0.76	53 ± 33		0.89 ± 0.86		21 ± 10	17 ± 12
	∆: −0.43 ± 4.84			∆: 0.00 ± 1.71	∆: −0.64 ± 3.25			∆: −0.31 ± 3.40				∆: +3 ± 28.33			∆: −1.15 ± 1.42	∆: −34 ± 39.66		∆: −1.07 ± 1.17		∆: −9 ± 17.20	∆: −4 ± 17.68
X	2.76 ± 0.93	1.79 ± 0.62	1.20 ± 0.54	6.82 ± 0.53	2.17 ± 0.67	5.65 ± 1.32	6.00 ± 0.67	1.09 ± 0.45	4.37 ± 1.60	4.58 ± 0.63	9.14 ± 0.66	22.87 ± 4.88	−7.00 ± 0.01	0.30 ± 0.33	4.11 ± 0.32	43.99 ± 4.08	0.43 ± 0.42	1.01 ± 0.62	0.05 ± 0.16	25.88 ± 10.47	27.68 ± 6.33
*D*	−1.16 ± 0.94	+1.19 ± 0.50	−1.58 ± 0.94	−0.02 ± 0.68	- 0.63 ± 1.08	−0.34 ± 1.49	−0.02 ± 0.98	−0.22 ± 0.69	−0.67 ± 2.24	−0.93 ± 0.67	−0.04 ± 0.93	−14.30 ± 6.82	+0.08 ± 0.01	−0.83 ± 0.65	−0.87 ± 0.51	−24.74 ± 5.17	−1.5 ± 0.74	−0.93 ± 0.82	−0.60 ± 0.38	−11.14 ± 13.15	−11.39 ± 11.45
6-month follow-up																					
Almohareb [30] Group 1 n. 20	3.24 ± 1.52	2.64 ± 1.23	3.12 ± 1.09									21.8 ± 9.1			4.4 ± 1.1	27.2 ± 13.3					
	∆: −2.49 ± 1.89	∆: −1.58 ± 2.12	∆: −1.07 ± 2.21									∆: −16.8 ± 13.15			∆: −0.8 ± 2.28	∆: −18.1 ± 19.9					
Group 2 n. 20	3.96 ± 1.11	2.98 ± 1.18	3.41 ± 0.89									20.1 ± 7.7			4.7 ± 1.0	29.7 ± 13.2					
	∆: −1.33 ± 1.98	∆: −1.48 ± 1.69	∆: −1.13 ± 1.40									∆: −21.1 ± 14.01			∆: −0.7 ± 2.32	∆: −14.1 ± 19.18					
Bombeccari [31] Group 1 n. 20										1.77			−6.57 ± 0.02		4.90 ± 0.47	10			0.00 ± 0.00		
										∆: −0.58 ± 0.02			∆: +0.54 ± 0.02		∆: −1.00 ± 0.89	∆: −60			∆: −0.70 ± 0.00		
Group 2 n. 20										2.06			−6.95 ± 0.03		5.50 ± 0.52	50			0.30 ± 0.48		
										∆: −0.31 ± 0.03			∆: +0.10 ± 0.04		∆: −0.30 ± 0.94	∆: −30			∆: −0.30 ± 0.70		
Chen [32] Group 1 n. 13										8.80 ± 2.49					4.10 ± 2.12	47.33					
										∆: −0.43 ± 3.95					∆: −0.85 ± 2.73	∆: −14.17					
Group 2 n. 12										8.66 ± 2.55					3.23 ± 1.89	44.33					
										∆: −3.36 ± 3.18					∆: −0.42 ± 2.39	∆: −14.00					
Laleman [34] Group 1 n. 9	5.21 ± 3.13			6.68 ± 1.23	1.06 ± 2.11			2.44 ± 2.41				2 ± 6			4.15 ± 0.96	59 ± 32		0.89 ± 0.63		20 ± 12	16 ± 6
	∆: +0.08 ± 4.43			∆: −0.25 ± 1.46	∆: −1.40 ± 2.89			∆: −0.65 ± 3.50				∆: −13 ± 14.32			∆: −1.02 ± 1.33	∆: −28 ± 34.53		∆: −1.03 ± 0.94		∆: −9 ± 16.28	∆: −14 ± 11.66
Group 2 n. 10	3.10 ± 3.48			6.90 ± 1.25	2.02 ± 2.19			2.45 ± 2.92				7 ± 14			4.18 ± 1.26	53 ± 39		1.22 ± 1.07		21 ± 11	17 ± 11
	∆: −0.41 ± 4.84			∆: + 0.03 ± 1.54	∆: −0.02 ± 3.16			∆: −1.29 ± 3.83				∆: −1 ± 25.24			∆: −1.27 ± 1.74	∆: −34 ± 44.78		∆: −0.74 ± 1.33		∆: −9 ± 17.80	∆: −4 ± 17.03
X	3.76 ± 0.66	2.81 ± 0.85	3.27 ± 0.69	6.76 ± 0.88	1.54 ± 1.53			2.45 ± 1.91		4.53 ± 0.69		15.70 ± 3.79	−6.76 ± 0.03		4.53 ± 0.41	36.66 ± 4.38		0.98 ± 0.64	0.15 ± 0.01	20.53 ± 8.11	16.53 ± 5.96
*D*	−0.16 ± 0.82	−0.17 ± 0.27	+0.49 ± 0.79	−0.05 ± 0.98	- 1.26 ± 1.69			+ 1.14 ± 0.10		−0.98 ± 0.70		−21.47 ± 6.08	+0.32 ± 0.02		−0.45 ± 0.57	−32.07 ± 5.41		−0.96 ± 0.83	−0.50 ± 0.35	−16.49 ± 11.36	−22.54 ± 11.25
12-month follow-up																					
Almohareb [30] Group 1 n.20	4.67 ± 1.44	3.33 ± 1.74	3.75 ± 1.79									25.6 ± 8.0			3.8 ± 0.9	18.6 ± 7.9					
	∆: −1.06 ± 1.82	∆: −0.89 ± 2.45	∆: −0.44 ± 2.62									∆: −13.0 ± 12.42			∆: −1.4 ± 2.19	∆: −26.7 ± 16.77					
Group 2 n.20	4.48 ± 1.35	3.86 ± 1.89	3.96 ± 1.88									27.1 ± 9.3			4.1 ± 1.0	25.7 ± 8.1					
	∆: −0.81 ± 2.12	∆: −0.60 ± 2.24	∆: −0.58 ± 2.17									∆: −14.1 ± 14.95			∆: −1.3 ± 2.32	∆: −18.1 ± 16.09					
X	4.58 ± 3.24	3.60 ± 1.82	3.86 ± 1.84									26.35 ± 4.28			3.95 ± 0.32	22.15 ± 5.66					
*D*	+0.66 ± 3.24	+0.62 ± 2.14	+1.08 ± 2.36									−10.82 ± 6.40			−1.03 ± 0.51	−46.58 ± 6.49					

Abbreviations: number, “n.”; logarithm, “log”; Colony-Forming Unit, “CFU”; milliliters, “mL”; millimeters, “mm”; percentage, “%”; deviation from baseline, “∆”; weighted average, “X”; mean deviation from baseline, “*D*”; plus or minus sign, “±“; *Porphyromonas gingivalis*, “P.g.”; *Tannarella forsythia*, “T.f.”; *Treponema denticola*, “T.d.”; *Fusobacterium nucleatum*, “F.n.”; *Prevotella intermedia*, “P.i.”; *Peptostreptococcus micros***,** “P.m.”; *Campylobacter rectus***,** “C.r.”; *Aggregatibacter actinomycetemcomitans***,** “A.a.”; *Eikenella corrodens*, “E.c.”; Plaque Index, “PI”; Clinical Attachment Level, “CAL”; modified Plaque Index, “mPI”; Probing Depth, “PD”; Bleeding on Probing, “BoP”; Papilla Bleeding Index, “PBI”; modified Sulcus Bleeding Index, “mSBI”; Suppuration on Probing, “SoP”; Full Mouth Plaque Score, “FMPS”, Full Mouth Bleeding Score, “FMBS”.

**Table 3:** Risk of bias assessment for the six RCTs included using the RoB 2 tool [29].

	Randomization Process (Domain 1)			Effect of Assignment to Intervention (Domain 2)							Effect of Adhering to Intervention (Domain 3)						Missing Outcome Data (Domain 4)				Measurement of the Outcome (Domain 5)					Selection of the Reported Studies (Domain 6)		
	1.1	1.2	1.3	2.1	2.2	2.3	2.4	2.5	2.6	2.7	3.1	3.2	3.3	3.4	3.5	3.6	4.1	4.2	4.3	4.4	5.1	5.2	5.3	5.4	5.5	6.1	6.2	6.3
Almohareb et al. [30]	Y	PN	N	NI	PY	N	N	NA	Y	NA	NI	PY	N	N	N	Y	NI	PN	PN	NI	PN	N	N	NA	NA	Y	N	N
Birang et al. [35]	Y	PY	NI	N	N	NA	NA	NA	Y	NA	N	N	NA	N	N	NA	Y	NA	NA	NA	NI	N	NI	NI	N	Y	N	N
Bombeccari et al. [31]	NI	PN	N	PY	PY	N	N	NA	PY	NA	PY	PY	N	N	N	Y	NI	PN	PN	NI	PY	N	N	NA	NA	Y	N	N
Chen et al. [32]	Y	Y	N	NI	NI	NA	N	NA	Y	NA	NI	NI	NA	N	N	NA	Y	NA	NA	NA	NI	N	NI	PN	N	Y	N	N
Galofré et al. [33]	NI	PN	N	N	N	NA	N	NA	Y	NA	N	N	NA	N	N	NA	PY	NA	NA	NA	NI	N	N	PY	PY	Y	NI	NI
Laleman et al. [34]	Y	Y	N	N	N	NA	N	NA	Y	NA	N	N	NA	N	N	NA	PY	NA	NA	NA	Y	N	NI	PY	N	Y	N	N

Abbreviations: Yes, “Y”; no, “N”; probably yes, “PY”; probably no, “PN”; not applicable, “NA”; no information, “NI”.

## Data Availability

Data are available in the MEDLINE/PubMed, Scopus, and BioMed. Central databases.

## Supplementary Materials

The following supporting information can be downloaded at: https://www.mdpi.com/article/10.3390/dj12120414/s1. Table S1: Peri-implantitis-associated microbiota variations over time, before and after peri-implantitis treatment.
