# Supplementary material for: Potential Impact of Microbial Variations After Peri-Implantitis Treatment on Peri-Implant Clinical, Radiographic, and Crevicular Parameters: A Systematic Review
Source: Dent J (Basel). 2024 Dec 17;12(12):414. doi: 10.3390/dj12120414 (PMC11674133; doi:10.3390/dj12120414)
Supplement: Supplementary file 1 [file dentistry-12-00414-s001.zip › dentistry-3300131-supplementary.pdf]

Potential Impact of Microbial Variations after Peri-implantitis Treatment on Peri-Implant Clinical, Radiographic, and Crevicular Parameters: A Systematic Review of Randomized Clinical Trials.

Dentistry Journal

Federica Di Spirito[1]*, Massimo Pisano[1]†, Maria Pia Di Palo[1]†, Flora Salzano[1], Antonio Rupe[1], Antonino Fiorino[2]‡, Carlo Rengo[1]*‡

[1]Department of Medicine, Surgery and Dentistry, University of Salerno, Via S. Allende, 84081 Baronissi, SA, Italy
[2]Department of Neuroscience, Reproductive Science and Dentistry, University of Naples Federico II, 80131 Naples, Italy

Corresponding Author: Federica Di Spirito (fdispirito@unisa.it); Carlo Rengo (crengo@unisa.it)

SUPPLEMENTARY FILE S1

Table S1. Peri-implantitis-associated microbiota variations over time before and after peri-implantitis treatment: bacterial counts (log CFU/mL) recorded at the baseline and 4-6 weeks and 3-6-12 months follow-up.

**Peri-implant microbial load and predominant pathogens' counts (log CFU/mL) (Treated) Peri-implantitis sites**								
**P.g.**	**T.f.**	**T.d.**	**F.n.**	**P.i.**	**P.m.**	**C.r.**	**A.a.**	**E.c.**
**Before peri-implantitis treatment (baseline)**								
3.92 ± 0.13	2.98 ± 1.12	2.78 ± 1.48	6.84 ± 0.42	2.80 ± 0.71	5.99 ± 0.70	6.02 ± 0.72	1.31 ± 0.52	5.04 ± 1.57
**4 weeks follow-up**								
5.25 ± 2.27	5.07 ± 1.72	3.89 ± 3.19	6.10 ± 2.14	6.43 ± 1.54	5.05 ± 2.21	5.31 ± 1.63	0.00 ± 0.00	4.74 ± 1.77
**6 weeks follow-up**								
4.38 ± 2.29			6.71 ± 0.81	1.88 ± 1.66			0.44 ± 1.44	
**3 months follow-up**								
2.76 ± 0.93	1.79 ± 0.62	1.20 ± 0.54	6.82 ± 0.53	2.17 ± 0.67	5.65 ± 1.32	6.00 ± 0.67	1.09 ± 0.45	4.37 ± 1.60
**6 months follow-up**								
3.76 ± 0.66	2.81 ± 0.85	3.27 ± 0.69	6.76 ± 0.88	1.54 ± 1.53			2.45 ± 1.91	
**12 months follow-up**								
4.58 ± 3.24	3.60 ± 1.82	3.86 ± 1.84						

Abbreviations: logarithm, "log"; Colony Forming Unit, "CFU"; milliliters, "mL"; plus or minus sign, "±"; *Porphyromonas gingivalis*, "P.g."; *Tannarella forsythia*, "T.f."; *Treponema denticola*, "T.d."; *Fusobacterium nucleatum*, "F.n"; *Prevotella intermedia*, "P.i."; *Peptostreptococcus micros*, "P.m."; *Campylobacter rectus*, "C.r."; Ag*gregatibacter actinomycetemcomitans*, "A.a."; *Eikenella corrodens*, "E.c.".
